# miR‐126 downregulates *CXCL12* expression in intestinal epithelial cells to suppress the recruitment and function of macrophages and tumorigenesis in a murine model of colitis‐associated colorectal cancer

**DOI:** 10.1002/1878-0261.13218

**Published:** 2022-04-11

**Authors:** Shuai Wu, Wei Yuan, Weiwei Luo, Kai Nie, Xing Wu, Xiangrui Meng, Zhaohua Shen, Xiaoyan Wang

**Affiliations:** ^1^ Department of Gastroenterology The Third Xiangya Hospital The Central South University Changsha China; ^2^ Key Laboratory of Non‐resolving Inflammation and Cancer of the Hunan Province The Third Xiangya Hospital The Central South University Changsha China; ^3^ Department of Hepatology The First Affiliated Hospital The Hunan University of Chinese Medicine Changsha China

**Keywords:** colorectal cancer, CXCL12, intestinal epithelial cells, macrophages, miR‐126

## Abstract

Inflammatory bowel disease, characterised by chronic relapsing‐remitting colitis, is a significant risk factor for colorectal cancer (CRC). Previously, we showed that miR‐126 functions as a tumour suppressor in CRC and is inversely correlated with tumour proliferation, metastasis and patient prognosis. In the current study, we documented a protective role for miR‐126 in colitis‐associated CRC (CAC) and its underlying mechanism. We detected downregulated miR‐126 expression during colorectal tumorigenesis in the mouse CAC model and in specimens from patients with CRC. The deficiency of miR‐126 in intestinal epithelial cells (IECs) exacerbated tumorigenesis in mice. We identified CXCL12 as a direct target of miR‐126 in inhibiting the development of colitis and CAC. Moreover, miR‐126 regulated the recruitment of macrophages via CXCL12 and decreased the levels of proinflammatory cytokines (IL‐6, IL‐12 and IL‐23). In addition, IL‐6 secreted by macrophages, which were regulated by cocultured transfected CRC cells, altered the proliferation and migration of colon cells. Our data suggest that miR‐126 exerts an antitumour effect on CAC by regulating the crosstalk between IECs and macrophages via CXCL12‐IL‐6 signalling. Our study contributes to the understanding of cancer progression and suggests miR‐126 as a potential therapy for CRC.

## Graphical abstract

In this study, we demonstrated that miR‐126 targets *CXCL12* in IECs, leading to mRNA cleavage or translational repression and therefore the inhibition of CXCL12 production. This reduction in CXCL12 expression subsequently inhibits macrophage functions, including migration to the tumour site and secretion of the proinflammatory cytokine IL‐6, to suppress colitis and tumorigenesis.

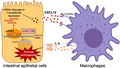

**Abbreviations**
AOM: azoxymethane
CAC: colitis‐associated colorectal cancer
CD: Crohn’s disease
CRC: colorectal cancer
DAI: disease activity index
DSS: dextran sulfate sodium
EMT: epithelial‐mesenchymal transition
H&E: haematoxylin and eosin
IBD: inflammatory bowel disease
IECs: intestinal epithelial cells
IHC: immunohistochemical
ISH: *in situ* hybridization
LPL: lamina propria lymphocytes
RISC: RNA‐induced silencing complex
TAMs: tumour‐associated macrophages
UC: ulcerative colitis

## 1 Introduction

Colorectal cancer (CRC) is the third most prevalent malignant tumour worldwide and ranks second in mortality [1]. Colorectal carcinogenesis is a multistep process (from normal mucosa to overt cancer) encompassing genetic and epigenetic aberrations and arises through distinct carcinogenic pathways: the classic adenoma‐carcinoma sequential pathway, serrated pathway and inflammatory pathway [2]. The inflammatory pathway involving chronic inflammation accelerates the clonal evolution of mutant cells and the development of overt neoplasia through repeated cycles of epithelial wounding and repair and leads to colitis‐associated cancer (CAC) [3].

Characterised by chronic relapsing‐remitting inflammation of the gastrointestinal tract, inflammatory bowel disease (IBD), including Crohn’s disease (CD) and ulcerative colitis (UC), is a significant risk factor for CAC [4]. For example, in patients with IBD, particularly patients with UC, a 2.4‐fold higher risk of developing CRC (95% confidence interval, 2.1–2.7) was reported in a meta‐analysis based on the data from 10 385 patients from eight cohort studies during 7–24 years (mean 14 years) of follow‐up [5]. IBD‐associated CRC appears similar to sporadic CRC and proceeds via the gradual stepwise accumulation of a series of genetic alterations in the normal colonic epithelium, such as mutations in *APC*, *KRAS* and *TP53*, and epigenetic modifications [including microRNAs (miRNAs), long noncoding RNAs, DNA methylation and histone modifications] that result in the development of CAC [2, 6]. However, the mechanisms by which the accumulated epigenetic modifications in intestinal epithelial cells (IECs) affect the development of CAC remain elusive.

miRNAs are a class of small noncoding RNA molecules with a size of 20–25 nucleotides. By binding to the mRNAs at the 3′‐untranslated regions (UTRs) through complementary base‐pairing, miRNAs are incorporated with the Argonaute (Ago) proteins into the RNA‐induced silencing complex (RISC), thereby inducing the translational repression or deadenylation and degradation of their target mRNAs [7]. miRNAs play essential roles in maintaining gut homeostasis and regulating various pathophysiological conditions, including the development of inflammatory diseases and cancer [8, 9]. During inflammation, miRNA function is generally suppressed, and impairment of global miRNA function, resembling obligate haploinsufficiency of Dicer, enhances inflammation‐associated tumorigenesis in a mouse colitis‐induced tumour model [10]. However, despite much attention drawn on the regulating effect of specific miRNAs on intestinal inflammation and CAC, the underlining mechanisms are not fully understood yet.

As previously shown in both human and mouse studies reported by our group and other researchers, miR‐126 functions as a tumour suppressor in several cancer types, including colon cancer [11, 12, 13, 14, 15, 16, 17, 18]. We found that miR‐126 expression is downregulated in colon cancer tissues compared with paraneoplastic distal colon mucosal tissues and inversely correlated with the clinical stage, lymph node metastasis and overall survival of patients with colon cancer [14, 17]. Furthermore, transfection of miR‐126 mimics inhibits the growth, cell‐cycle progression, migration and invasion of colon cancer cells *in vitro* [14, 15]. We further investigated the role of miR‐126 in CRC using nude mice that were subcutaneously or intravenously injected with colon cancer cells overexpressing or silencing miR‐126, and the findings revealed that miR‐126 inhibits the tumorigenicity and metastasis of colon cancer cells *in vivo* as well [17]. Nevertheless, researchers have not determined whether miR‐126 modulates immune cells in inflammatory foci and during colitis‐associated tumorigenesis.

During the initiation and progression of CAC, chronic inflammation is controlled by a complex interaction of infiltrated immune cells and associated disequilibrium between proinflammatory and anti‐inflammatory cytokines [19, 20]. Single‐cell analyses of inflamed tissues from patients with IBD revealed a module comprising inflammatory macrophages, stromal cells, T and B lymphocytes and activated dendritic cells that construct the cell–cell interaction network in the mucosal milieu [21, 22]. As one of the dominant cell types in the inflammatory milieu, inflammatory macrophages produce a range of chemokines (CXCL2, CXCL3 and CXCL8) and proinflammatory cytokines [IL‐6, IL‐1β, IL1α, tumour necrosis factor‐alpha (TNF‐α) and IL‐23] [23]. These signals modulate IEC activity by affecting proliferation, migration and survival programmes, and creating a microenvironment conducive to epithelial transformation and ultimately tumour development [24, 25].

In the current study, we investigated the role of miR‐126 in CAC and its effect on the macrophage–IEC cell interaction. We established a murine CAC model to analyse the miR‐126 expression profile during the ‘normal‐inflammation‐dysplasia‐carcinoma’ sequential tumorigenesis process. We generated IEC‐specific miR‐126‐deficient (miR‐126^ΔIEC^, cKO) mice and modelled CAC to determine the function of miR‐126. As algorithm predictions and RNA immunoprecipitation assays identified CXCL12 as a direct target of miR‐126, we investigated the effects of CXCL12 on miR‐126^ΔIEC^ mice during CAC development and on regulating the proportion of macrophages and cytokine levels. Furthermore, as we found that miR‐126 regulated the recruitment of macrophages and inhibited proinflammatory cytokine production via CXCL12, we established two‐step coculture systems containing IECs and macrophages. The results indicated that the crosstalk between these cells might suppress epithelial transformation and ultimately inhibit tumour development.

## 2 Materials and methods

### 2.1 Subjects

Intestinal specimens were obtained from 12 patients with CRC, 6 with UC, 9 with CD and 10 non‐IBD/CRC patients who underwent surgical resection in the Third Xiangya Hospital during a 2‐year period (2019–2021), except for patients with UC, which included a 5‐year period (2016–2021). The diagnosis was made based on haematoxylin and eosin (H&E) staining of both endoscopic biopsies and surgical specimens obtained during colonoscopy and surgical resection. The diagnosis of IBD was based on widely agreed upon clinical, endoscopic and histological criteria and the exclusion of infectious and systemic diseases. Subjects not diagnosed with IBD or CRC based on endoscopic and histopathologic findings were classified as the ‘control’ group, including patients enrolled because of traumatic intestinal rupture, intestinal obstruction and volvulus, excluding those diagnosed with IBD or indeterminate colitis or gastrointestinal cancer. Details of the clinical characteristics of the patients included in this study are summarised in Tables 1 and 2. The study was approved by the Institutional Review Board for Clinical Research of the Third Xiangya Hospital of Central South University and in accordance with the Helsinki Declaration. Written informed consent was also obtained from all subjects before the initiation of the study protocol.

**Table 1:** Clinical characteristics of patients in this study. Numeric data are summarised as mean ± SD, with the range given by [min–max].

	Non‐IBD	UC	CD	CRC
Subjects	10	6	9	12
Sex				
Female	2	3	2	8
Male	8	3	7	4
Age (years)	46.7 ± 20.4	42.7 ± 10.8	34.2 ± 13.3	50.25 ± 13.4
	[22–77]	[25–56]	[20–57]	[29–71]
CD Montreal location				
L1				
L1 + L4				
L2				
L2 + L4				
L3			3	
L3 + L4			6	
UC extent				
Ulcerative proctitis				
Left‐sided UC		1		
Extensive UC/pancolitis		5		
Tumour location				
Left‐sided colon				5
Right‐sided colon				3
Sigmoid				4

**Table 2:** Characteristics of CRC patients.

No.	Age (years)	Sex	Location	Histological grade	TNM classification			Stage	MSI (PCR)	CDX‐2	SATB2	BRAF V600E	Ki67
					T	N	M						
1	59	Female	Sigmoid	2	4b	2a	1a	IVA	MSS	+	+++	−	50%+
2	67	Female	Left‐sided colon	2	3	0	1c	IVC	MSS	++	++	−	40%+
3	65	Male	Left‐sided colon	2	3	2a	0	IIIB	MSS	++	++	−	70%+
4	44	Female	Left‐sided colon	2	3	2a	0	IIIB	MSS	+	+	−	20%+
5	47	Female	Sigmoid	2	2	0	0	I	MSI‐H	+	+	−	20%+
6	37	Female	Sigmoid	2	2	1b	0	IIIA	MSS	+	+	−	20%+
7	50	Female	Sigmoid	2	3	0	0	IIA	MSS	+	+	−	60%+
8	71	Male	Left‐sided colon	2	3	1b	0	IIIA	MSS	+	+	−	40%+
9	29	Female	Right‐sided colon	3	3	1b	1a	IVA	MSS	+	−	−	60%+
10	33	Male	Left‐sided colon	3	3	1b	0	IIIB	MSS	+	−	−	20%+
11	49	Female	Right‐sided colon	2	3	0	0	IIA	MSI‐H	+	Partly+	−	80%+
12	52	Male	Right‐sided colon	3	3	1b	1a	IVA	MSS	+	+	−	60%+

### 2.2 Generation of IEC‐specific miR‐126‐deficient mice

miR‐126^ΔIEC^ mice on a C57BL/B6 background were generated using the Cre/loxP and Flp/FRT recombination systems (Shanghai Model Organisms Center, Shanghai, China). Briefly, miR‐126^flox‐Neo/+^ mice were generated by targeting exon 1 of miR‐126 using ES cell targeting methods. The miR‐126^flox‐Neo/+^ mice were then crossed with Flp transgenic mice [FLPo (B6 ROSA26Flpo), stock no. 012930; The Jackson Laboratory, Bar Harbour, ME, USA] to delete the Neo cassette and obtain miR‐126^fl/+^ mice, which were crossed with Villin‐Cre mice [Tg (Vil1‐Cre) mice, stock no. 0021504; The Jackson Laboratory] to generate miR‐126^ΔIEC^ mice (Fig. S1). All mice used in this study were maintained under specific pathogen‐free conditions and bred in‐house to generate comparable groups. All animal studies were conducted in strict accordance with the recommendations in the Guide for the Care and Use of Laboratory Animals. The protocol was approved by the Animal Ethics Committee of Central South University (No. 2018‐S092, December 2018).

### 2.3 Induction of CAC and scoring of the disease activity index

Eight‐ to ten‐week‐old male and female miR‐126^ΔIEC^ mice, their wild‐type (WT) littermates and C57BL/6 mice were intraperitoneally injected with azoxymethane (AOM, Cat. no. A5486; Sigma–Aldrich, St. Louis, MO, USA; 10 mg·kg^−1^; dissolved in physiological saline). Within each group, the numbers of male and female mice were roughly equal. One week later, the mice were provided drinking water containing 1.5% dextran sulfate sodium (DSS, Cat. no. 02160110‐CF, 36 000–50 000 M.Wt.; MP Biomedicals, Solon, OH, USA) for the next seven consecutive days, followed by regular water for 2 weeks. This cycle was repeated three times and then animals were provided regular water until the end of the experiment in the 18th week [26]. Mice were treated daily with AMD3100 (Cat. no. A5602; Sigma–Aldrich; 5 mg·kg^−1^, i.p.) to interfere with the CXCL12/CXCR4 axis or PBS for the control group. Mice were sacrificed at the indicated time intervals (at the end of the 0th, 2nd, 6th, 10th and 18th weeks). Colonic tissues were opened longitudinally, and colon size and the number of tumours were measured. Colon sections were frozen in liquid nitrogen for total RNA and protein extraction or fixed with 4% paraformaldehyde, dehydrated and embedded in paraffin for histological analyses. Serum collected from each mouse was prepared to measure cytokine levels. All mouse procedures were performed according to institutional guidelines.

The severity of colitis was assessed by determining the disease activity index (DAI) as previously described [27]. The DAI is the combined score of weight loss, stool consistency and bleeding. Scores were defined as follows: weight: 0, no loss; 1, 1–5%; 2, 5–10%; 3, 10–20%; and 4, > 20% weight loss; stool: 0, normal; 2, loose stool; and 4, diarrhoea; and bleeding: 0, no blood; 2, occult blood‐positive; and 4, gross blood (Hemoccult Single Slides; Beckman Coulter, Brea, CA, USA).

### 2.4 Histopathological analysis

Paraffin‐embedded intestinal specimens from patients and experimental mice were stained with H&E for the microscopic examination. The sections were reviewed and scored for inflammation by two pathologists who were blinded to the treatment based on the following criteria [28]: inflammatory cell infiltration (graded as 0 = no infiltration, 1 = mild mucosal infiltration, 2 = moderate mucosal and submucosal infiltration and 3 = marked transmural infiltration) was added to the intestinal architecture (0 = no ulceration, 1 = focal erosions, 2 = erosions ± focal ulcerations, 3 = extended ulcerations ± granulation tissue ± pseudopolyps) for a final score with six possible values.

### 2.5 *In* *situ* hybridisation and immunohistochemistry

The expression of miR‐126 in colonic tissues was detected by performing *in situ* hybridisation (ISH) on the mouse and human paraffin colonic sections using digoxigenin (DIG)‐labelled miRNA probes. Briefly, sections were treated with proteinase K (20 µg·mL^−1^, Cat. no. G1205; Servicebio Technology, Wuhan, China) for 20 min at 37 °C after dewaxing and dehydration, followed by prehybridisation at 37 °C for 1 h and hybridisation with 1 µm DIG‐labelled miR‐126 probes (5′‐DIG‐TCACTGTTGCCTGCTGAGATTA‐DIG‐3′) overnight at 42 °C. Stringent washes were performed with SSC buffers (Cat. no. G3016‐4; Servicebio Technology) at 37 °C. The sections were then incubated with a blocking reagent (rabbit serum, Cat. no. G1209; Servicebio Technology) and stained with anti‐DIG‐horseradish peroxidase (HRP) at 37 °C for 50 min, Cy3‐tyramine signal amplification (TSA) for 5 min and DAPI for 8 min at room temperature. All sections were mounted with anti‐fluorescence quenching mounting mediums (Cat. no. G1401; Servicebio Technology).

Immunohistochemistry (IHC) was performed on 4 µm‐thick paraffin‐embedded colorectal tissue sections from mice. The deparaffinised sections were incubated with a CXCL12 polyclonal antibody (Cat. no. 17402‐1‐AP; Proteintech, Rosemont, IL, USA; 1 : 200), a CD68 polyclonal antibody (Cat. no. 28058‐1‐AP; Proteintech; 1 : 1000), a Ly6G monoclonal antibody (Cat. no. 87048; Cell Signaling Technology, Danvers, MA, USA; 1 : 100), a CD4 monoclonal antibody (Cat. no. 25229; Cell Signaling Technology; 1 : 100), a CD8 monoclonal antibody (Cat. no. 66868‐1‐Ig; Proteintech; 1 : 16 000) or a PAX5 polyclonal antibody (Cat. no. 21383‐1‐AP; Proteintech; 1 : 200) at 4 °C overnight followed by incubation with a biotinylated goat anti‐rabbit or mouse IgG antibody (Vector Laboratories, Burlingame, CA, USA) for 30 min. The immunostained sections were observed under a microscope (OLYMPUS BX‐51, Tokyo, Japan) and scored based on the immunoreactive score system [29]: positive cells proportion score (graded as 0 = no positive cells, 1 = < 10% of positive cells, 2 = 10–50% positive cells and 3 = 51–80% positive cells, 4 = > 80% positive cells) was multiply by staining intensity score (graded as 0 = no reaction, 1 = mild reaction, 2 = moderate reaction and 3 = intense reaction) for a final score with 12 possible values.

### 2.6 Isolation of colonic lamina propria lymphocytes (LPLs) and flow cytometry analysis

The intestines were opened longitudinally and cut into 0.5 cm sections and shaken twice with HBSS containing 10 mm HEPES, 25 mm NaHCO_3_, 1 mm DTT, 1 mm EDTA (Cat. no. H8264; Sigma–Aldrich) and 2% fetal bovine serum (Gibco, Portland, OR, USA) at 37 °C for 20 min. IECs were then washed with HBSS to remove epithelial cells. For the isolation of LPLs, intestinal pieces were digested with complete RPMI‐1640 containing 1 mg·mL^−1^ collagenase D (Cat. no. 11088866001; Roche, Basel, Switzerland), 40 µL·mL^−1^ Dispase II (Cat. no. 4942078001; Sigma–Aldrich) and 4 µL·mL^−1^ DNase I (Cat. no.10104159001; Roche) for 40 min at 37 °C. The samples were then passed through a 70 µm cell strainer (BD Biosciences, Franklin Lakes, NJ, USA). The digestion step was repeated two or three times until no connective tissue was visible. Dissociated cells obtained from the digestion were washed twice with PBS, resuspended in 5 mL of 40% Percoll (Cat. no.17‐0891‐02; GE Healthcare, Boston, MA, USA), underlaid with 3 mL of 80% Percoll and centrifuged at 1000 ***g*** for 20 min at room temperature without braking. LP lymphocytes were recovered from the interphase of the Percoll gradient, washed twice and resuspended in FACS buffer (HBSS solution containing 3% FBS and 0.05% sodium azide) or RPMI medium [30].

Isolated LPLs were stained with PE‐conjugated anti‐mouse F4/80 antibodies (Cat. no. 565410; BD Biosciences) and FITC‐conjugated CD11b antibodies (Cat. no. 557396; BD Biosciences) or their respective fluorochrome‐conjugated isotype controls (Cat no. 553930 and Cat no. 553988; BD Biosciences) according to the manufacturer's instructions. Cells were analysed using a BD FACS CANTO II flow cytometer, and data were analysed using flowjo software (BD Biosciences).

### 2.7 LEGENDplex™ bead‐based immunoassays

The levels of IL‐6, IL‐12, IL‐23, IL‐1β, IL‐17A, TNF‐α, IL‐10 and transforming growth factor‐beta (TGF‐β) were measured in the serum from mice in each group at 0, 2, 6, 10 and 18 weeks after AOM/DSS treatment using LEGENDplex™ predefined mouse macrophage/microglia panel (Cat. no. 740848; Biolegend, San Diego, CA, USA) according to the manufacturer’s instructions. Briefly, a selected panel of capture beads (25 µL per well), which come in two sizes and exhibit different levels of APC fluorescence, are mixed and incubated with serum samples (12.5 µL per well, diluted 2‐fold with assay buffer) at room temperature for 2 h with shaking. After two washes, biotinylated detection antibodies (25 µL per well) were added. Each detection antibody binds to its specific analyte (in this case, the cytokines we analysed) that is bound to the capture beads, thus forming capture bead‐analyte‐detection antibody sandwiches. Streptavidin‐phycoerythrin (SA‐PE) was subsequently added (25 µL per well), which bound to the biotinylated detection antibodies, providing a fluorescent signal with intensities in proportion to the amount of bound analyte. The APC and PE signal fluorescence intensity of each bead population was quantified using a BD FACS CANTO II flow cytometer (BD Biosciences). The concentration of a particular analyte was determined based on a known standard curve using legendplex™ (Biolegend, San Diego, CA, USA) data analysis software.

### 2.8 Enzyme‐linked immunosorbent assay

Colonic tissues were snipped into small pieces with scissors and then subjected to homogenisation in 2 mL Eppendorf tubes for 60 s using 3 mm metal balls in a tissue‐grinder mill (Cat. no. KZ‐III; Servicebio Technology). Tubes were centrifuged, and the supernatant was collected to perform enzyme‐linked immunosorbent assay (ELISA). The levels of IL‐6, IL‐12, IL‐23 and CXCL12 in the colonic tissues were measured using commercial kits (Cat. no. MOES00663, MOES01221 and MOES01226; 4A Biotech, Beijing, China) and SDF‐1 alpha/CXCL12 Mouse ELISA Kit (Cat. no. EMCXCL12; Thermo Fisher Scientific Inc, Waltham, MA, USA), respectively, according to the manufacturer’s instructions.

### 2.9 Computationally predicted miRNA–mRNA interaction pairs

miRNA–mRNA interaction pairs were identified based on the miRNA‐target interactions generated by miRWalk2.0 [31], miRTarBase [32] and miRTargetLink Human [33] databases. The miRTarBase and miRTargetLink Human databases provide experimentally validated miRNA‐target interactions while the miRWalk2.0 database provides computationally predicted and experimentally validated miRNA‐target interactions. The miR‐126‐target interactions are listed in Tables [Link], [Link], [Link], respectively. The overlapped target mRNAs were filtered out using Venn diagrams and their details from The Human Protein Atlas [34] are described in Table S4. The hsa‐miR‐126‐3p‐*CXCL12* or mmu‐miR‐126‐3p‐Cxcl12 potential binding sites were predicted using miRanda and TargetScan [35], respectively.

### 2.10 Analysis of infiltrating immune cells

Using the comprehensive web resource TIMER, a systematic evaluation of immune infiltrations across different cancer types [36], we investigated the immune infiltrates abundance, including macrophages, neutrophils, CD4+ T cells, CD8+ T cells and B cells in colon adenocarcinoma (COAD). We further confirmed the correlations between the somatic copy number alteration of CXCL12 and the abundance of immune infiltrates using the SCNA module of TIMER.

### 2.11 Cell culture and transfection

Human CRC cell lines (Caco2, HT29 and HCT116), the HEK293 cell line and the human monocytic cell line THP‐1 were obtained from the Cancer Research Institute of Central South University. The detailed profile of those cells been used in this study are summarised in Table S5. All cells were cultured at 37 °C in RPMI‐1640 or DMEM (HyClone|Cytiva, Marlborough, MA, USA) supplemented with 10% fetal calf serum, 100 U·mL^−1^ penicillin and 100 mg·mL^−1^ streptomycin in a humidified atmosphere containing 5% CO_2_. THP‐1 cells (4 × 10^5^ mL^−1^) were differentiated into macrophages using 150 nm phorbol 12‐myristate 13‐acetate (PMA, Cat. no. P8139; Sigma–Aldrich) for 2 days. The miR‐126 mimic, miR‐126 inhibitor, corresponding scrambled negative control (NC) vectors and inhibitor negative control (iNC) vectors were synthesised by GenePharma (Suzhou, Jiangsu, China). Caco2 CRC cells were transfected with 50 nm miRNA mimics, inhibitors, NC or iNC vectors using Lipofectamine 3000 (Cat. no. L3000015; Thermo Fisher Scientific Inc) according to the manufacturer's instructions.

### 2.12 Two‐step coculture system of macrophages and colon cells

A two‐step coculture system of macrophages and colon cells was built. On Day 0, Caco2 cells (1 × 10^5^ cells/well) transfected with miRNA mimics, inhibitors, NC or iNC vectors were seeded in the bottom chamber in each well of 24‐well plates containing transwell inserts (8.0 μm pore; Merck Millipore, Burlington, MA, USA), and THP‐1‐induced macrophages (2 × 10^5^ cells/well) were seeded in the top chambers of the transwell inserts. After 2 days of coculture, the transwell inserts containing macrophages were removed and placed into another 24‐well plate in which HT29 or HCT116 cells (1 × 10^5^ cells/well) were seeded 24 h before this movement. A neutralising monoclonal antibody against human IL‐6 (Cat. no. mabg‐hil6‐3; InvivoGen, Hong Kong, China, 100 ng·mL^−1^) was added to the ‘anti‐miR‐126+IL‐6 Abs’ group.

### 2.13 Cell migration assays

The chemotactic effect of CXCL12‐ and miR‐126‐overexpressing or miR‐126‐silenced colon cells on macrophages was determined by performing transwell migration assays. Briefly, THP‐1‐induced macrophages (2 × 10^5^ cells/well) were cultured in 200 μL of FBS‐free medium in triplicate in the top chambers of transwell inserts in 24‐well plates (8.0 μm pore; Merck Millipore). Medium supplemented with 15% FBS was added to the bottom chambers (600 μL per chamber) along with 100 ng·mL^−1^ recombinant human CXCL12(Cat. no. 300‐28B; PeproTech, Cranbury, NJ, USA), 100 ng·mL^−1^ AMD3100 or Caco2 cells (transfected with miR‐126 mimics, NC, inhibitor or iNC) as chemotactic signals. After 24 h of culture, the macrophages remaining in the top chambers were removed with a cotton swab. The cells that migrated to the bottom of the transwell inserts were fixed with 4% paraformaldehyde, stained with a 0.1% crystal violet solution and imaged under a light microscope. Randomly selected cells in 10 fields were counted in a blinded manner. HCT116 cells were examined following the same procedure adding 20 ng·mL^−1^ recombinant human IL‐6 (Cat. no. 200‐06; PeproTech) to determine the chemotactic effect of IL‐6 on colon cells.

### 2.14 Wound healing assays

HCT116 cells were seeded in 6‐well plates (4 × 10^5^ cells per well) in triplicate, and a cross‐shaped scratch was made in the middle of each well with a 10 μL pipette tip. After incubating in a serum‐free medium for 56 h, the cells were gently rinsed with PBS to remove debris. Photographs were taken to estimate the closure of the gap in at least three randomly selected fields. Wound closure was then calculated using the formula as previously described [36]: wound closure (%) = (area of gap at the start point − area of gap at the endpoint)/area of gap at the start point. Each experiment was repeated at least three times.

### 2.15 Cell counting kit‐8 assays

HCT116 colon cancer cells, which were cocultured with ‘educated’ macrophages in the two‐step coculture system for 2 days, were digested and seeded in flat‐bottom 96‐well plates at a density of 1 × 10^4^ cells/well in quintuplicate. On Days 1, 2, 3 and 4 after seeding, 10 µL of cell counting kit (CCK)‐8 reagent (Cat. no. CK04; Dojindo, Kamimashiki‐gun, Kumamoto, Japan) were added to each well, and the cells were incubated at 37 °C for 2 h. The optical density (OD) was determined using a Varioskan LUX multimode microplate reader (Cat. No. VL0000D0; Thermo Fisher Scientific Inc.) at the wavelength of 450 nm.

### 2.16 RNA extraction and quantitative real‐time PCR

Total RNA was extracted from the cells and tissues using TRIzol reagent. The purity and concentration of RNA were determined using ultraviolet spectrophotometry. RNA integrity was assessed by performing agarose gel electrophoresis. Total RNA was used to synthesise cDNA templates by reverse transcription using the SuperScript™ RT reagent Kit (Cat. no. 11904018; Thermo Fisher Scientific Inc.) with a reaction system volume of 20 μL. Expression of the mRNAs was evaluated using quantitative real‐time PCR (qRT–PCR) with SYBR Premix Ex Taq kit (Cat. no. RR420A; Takara Biotechnology, Shiga, Japan) according to the standard protocol. GAPDH was used as an internal control and the relative quantitative method was applied to calculate the relative mRNA copy number (measured in triplicate). The $2^{‐\Delta \Delta C_{t}}$ method was used to calculate the ratio of target mRNA expression relative to GAPDH mRNA expression. The miDETECT A Track™ miRNA qRT–PCR kit (Cat. no. C10712‐2; RiboBio, Guangzhou, China) was used to synthesise the cDNA template of miR‐126 and analyse its expression. U6 small nuclear RNA was used as an internal control for the analysis of miRNA expression levels and the expression of each gene was quantified by measuring cycle threshold (*C*_t_) values and normalised using the $2^{‐\Delta \Delta C_{t}}$ method relative to U6 small nuclear RNA. for the following primer sequences were used: *Cxcl12* forward ACACTCCAAACTGTGCCCTT, reverse CTGTA AGGGTTCCTCAGGCG; *Gapdh* forward TGGATTTGGACGCATTGGTC, reverse TTTGCACTGGT ACGTGTTGAT; *miR‐126* forward GGGTCGTACCGTGAGTAAT, reverse GGGCATTATTACTTTTGG; and *U6* forward ATTGG AACGATACAGAGAAGATT, reverse GGAACGCTTCACGAATTTG.

### 2.17 RNA immunoprecipitation with anti‐pan‐Ago

For each sample, RNA from 2 × 10^7^ HEK293 cells was subjected to immunoprecipitation using the miRNA Target IP kit (Cat. no. 25500; Active Motif, Carlsbad, CA, USA) according to the manufacturer’s protocol. Briefly, approximately 24 h after the transfection of 5 μmol mimics‐miR‐126, cells were washed with ice‐cold PBS, scraped from the plate and lysed with 150 μL of ice‐cold complete lysis buffer (provided in the kit). Approximately 100 μL of the whole‐cell extract were incubated with 900 μL of lysis buffer. Immunoprecipitation buffer containing magnetic beads conjugated with a human anti‐pan‐Ago antibody or negative control normal mouse IgG (provided in the kit) was mixed with the samples and rotated overnight at 4 °C. Samples were washed seven times with 1× Wash Buffer. The samples were then incubated with proteinase K in buffer at 55 °C for 30 min with shaking to digest the protein. Coimmunoprecipitated RNA, including miRNA:mRNA complexes, was subjected to qRT–PCR analysis. According to the manufacturer’s protocol, the enrichment of miRNAs was calculated as follows: Fold Enrichment = AE (Neg IgG CT–Ago CT), where AE = Amplification Efficiency = 10(−1/slope) = 10(−1/−3.508). GAPDH served as an internal control to calculate the ratio of target mRNA expression relative to GAPDH mRNA expression. Representative bar diagrams were constructed from three independent experiments, and each set of experiments was performed in triplicate.

### 2.18 Western blotting

Total proteins were extracted from the cultured cells using RIPA buffer containing phosphatase and protease inhibitors (Roche). Equal aliquots of 50 μg of total proteins were separated on SDS/PAGE gels (10% acrylamide) and then transferred to PVDF membranes (Merck Millipore). Membranes were blocked with TBST containing 5% skim milk and then incubated overnight at 4 °C with a monoclonal anti‐CXCL12 antibody (Cat. no. 3530; Cell Signaling Technology; 1 : 1000), monoclonal anti‐IL‐6 antibody (Cat. no. 66146‐1‐Ig; Proteintech; 1 : 1000) or monoclonal anti‐GAPDH antibody (Cat. no. 60004‐1‐Ig; Proteintech; 1 : 10 000), followed by incubation with a goat anti‐mouse IgG‐HRP secondary antibody (Cat. no. sc2005; Santa Cruz Biotechnology, Dallas, TX, USA; 1 : 5000) or a mouse anti‐rabbit IgG‐HRP secondary antibody (Cat. no. sc2357; Santa Cruz Biotechnology; 1 : 5000) for 1 h at 37 °C. Finally, an ECL detection system (Merck Millipore) was used to detect signals.

### 2.19 Immunofluorescence staining

HT29 colonic cells were seeded in chamber slides and cocultured with macrophages that had been cocultured with miR‐126‐overexpressing or miR‐126‐silenced colon cells for 2 days. IL‐6 neutralising antibodies were added to the system. After 48 h, slides containing HT29 cells were removed. Cells were fixed with 4% paraformaldehyde for 20 min at room temperature. The cells were then thoroughly washed three times with 1× PBS and permeabilised with 0.1% Triton X‐100 (Cat. no. X100; Sigma–Aldrich) for 10 min. Cells were blocked with 5% FBS for 1 h followed by overnight incubation with a monoclonal anti‐Vimentin antibody (Cat. no. s6260; Santa Cruz Biotechnology; 1 : 50) or monoclonal anti‐E‐cadherin antibody (Cat. no. sc8426; Santa Cruz Biotechnology; 1 : 50) at 4 °C. Next, a goat anti‐mouse IgG – Alexa Fluor 488 or – Cy3 secondary antibody (Cat. No. GB25301 and GB21301; Servicebio Technology) was added to the cells and incubated for 1 h in the dark at room temperature.

### 2.20 Statistics

Data are presented as the mean ± standard deviations (SD) or mean ± standard errors of the mean (SEM) from at least three independent experiments. Graphical analyses and statistical analyses of the data were performed using prism software (GraphPad Software, San Diego, CA, USA). Differences between groups were determined using a two‐way analysis of variance (ANOVA) or the unpaired Student’s two‐tailed *t* test. Correlations were determined by Pearson’s correlation test. A *P* value < 0.05 was considered statistically significant.

## 3 Results

### 3.1 miR‐126 expression is downregulated during colitis and colorectal tumorigenesis

The well‐established mouse model of AOM/DSS‐induced CAC that is generated by chemical induction of DNA damage followed by repeated cycles of colitis provides a unique opportunity to examine the roles of miRNAs and immune cells in colon carcinogenesis [37]. AOM is a procarcinogen molecule that is metabolised by cytochrome p450 and converted to methylazoxymethanol (MAM) in the liver, which is a highly reactive alkylating species that induces the formation of O6 methylguanine adducts in DNA, resulting in G→A transitions. After excretion into the bile, MAM is taken up by the colonic epithelium and induces mutagenesis. DSS is a heparin‐like polysaccharide that inflicts colonic epithelial damage and induces colitis [38]. Combining AOM and repeated treatment with DSS produces a pathology characterised by severe colitis and body weight loss and bloody diarrhoea followed by the development of colon tumours. This two‐step CAC model represents a ‘normal – inflammation – low‐grade dysplasia – high‐grade dysplasia – carcinoma’ sequential pathological process, modelling chronic colitis and CAC in humans [26].

**Fig. 1:**
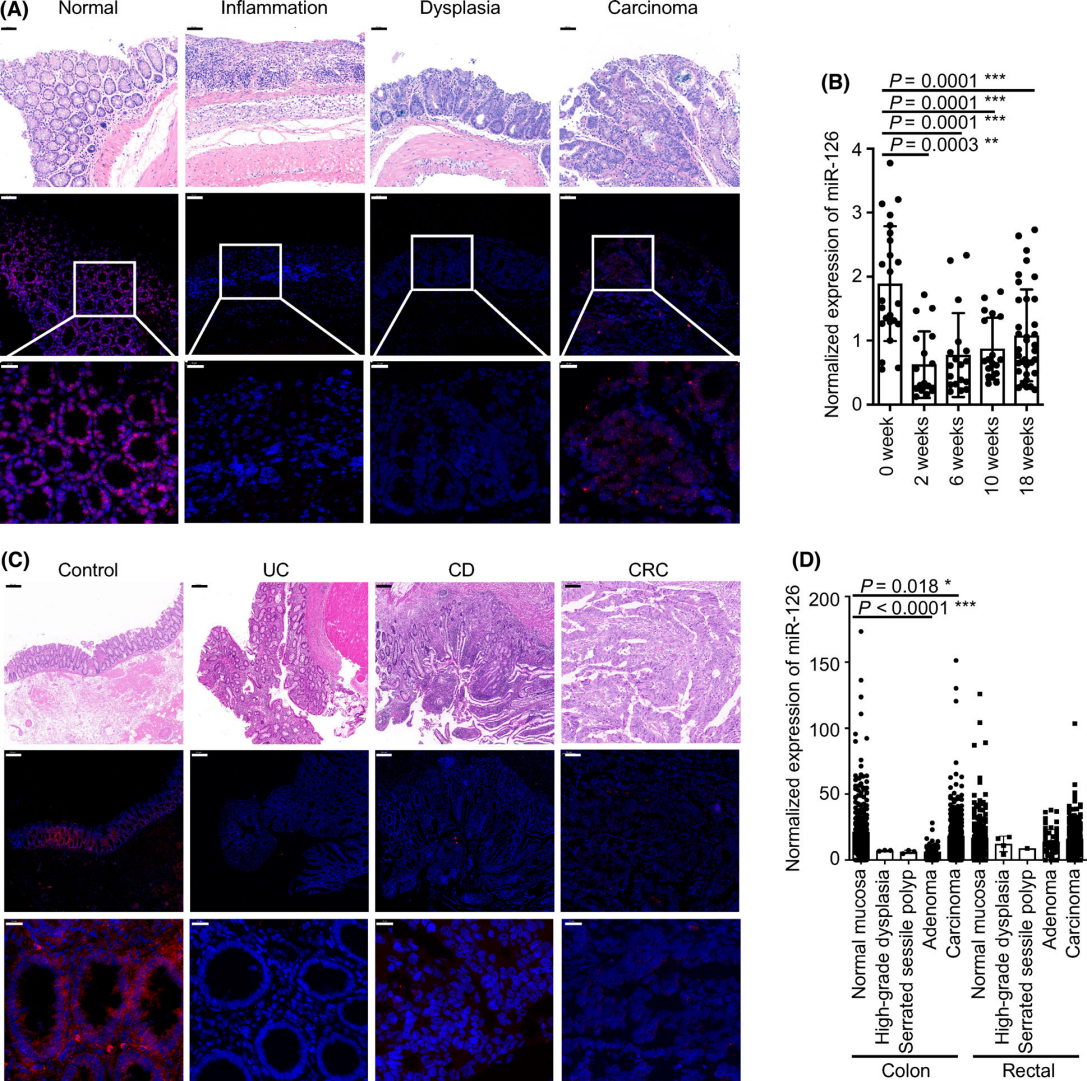
miR‐126 expression is downregulated during colitis and colorectal tumorigenesis. (A) Representative intestinal sections were prepared from normal colonic mucosal, inflamed mucosal, dysplasia and carcinoma tissues from AOM/DSS‐treated WT mice and subjected to miR‐126 ISH. Magnification: 200×, scale bar = 50 μm, upper panels; 600×, scale bar = 20 μm, lower panels (*n* = 5). (B) Relative expression of miR‐126 in colonic tissues from AOM/DSS‐treated WT mice at the 0th, 2nd, 6th, 10th and 18th weeks (*n* = 24, 18, 18, 18 and 36, respectively). (C) Representative intestinal sections were prepared from the colonic mucosa of a non‐IBD/CRC patient (Control), the inflamed mucosa of a patient with UC, a patient with CD and the carcinoma mucosa of a patient with CRC and subjected to miR‐126 ISH. Magnification: 200×; scale bar = 50 μm, upper panels: 600×; scale bar = 20 μm middle and lower panels (*n* = 10, 6, 9 and 12, respectively). (D) The expression of miR‐126 in colonic tissues was analysed in the GSE115513 microarray dataset, including 381 normal mucosa, 3 high‐grade dysplasia, 3 serrated sessile polyps, 51 adenomas and 411 carcinomas from colonic tissues and 268 normal mucosa, 4 high‐grade dysplasia, 1 serrated sessile polyp, 52 adenomas and 339 carcinomas from rectal tissues. Individual data are presented as the mean ± SD. Statistical analyses were performed using multiple unpaired *t* tests (*P < 0.05; **P < 0.01; and ***P < 0.0001).

Therefore, we collected colonic tissues from AOM/DSS‐treated mice at the indicated time points (at the 0th, 2nd, 6th, 10th and 18th weeks) to analyse the expression of miR‐126, and these time points approximated the five stages (normal, acute inflammation, chronic inflammation, dysplasia and carcinoma) of the inflammation‐cancer sequence. We detected significantly decreased miR‐126 expression in the inflamed, dysplastic and carcinoma tissues (Fig. 1A) and from colonic tissues collected at the 2nd, 6th, 10th and 18th weeks after treatment compared with untreated tissues (Fig. 1B). Moreover, miR‐126 expression was decreased in tissues from patients with UC and CD, especially in CAC tissues (Fig. 1C, Fig. S2A,B). In addition, data from the GSE115513 database, which contained expression profiles of 1893 carcinoma/normal paired human samples and 290 adenoma tissue samples [39], showed a decrease in miR‐126 expression in colon adenoma and carcinoma tissues compared with normal mucosal tissues (Fig. 1D). Based on these findings, miR‐126 expression was decreased during colorectal tumorigenesis either in a mouse model or in specimens from patients with CRC.

### 3.2 miR‐126 deficiency in IECs exacerbates CAC development

**Fig. 2:**
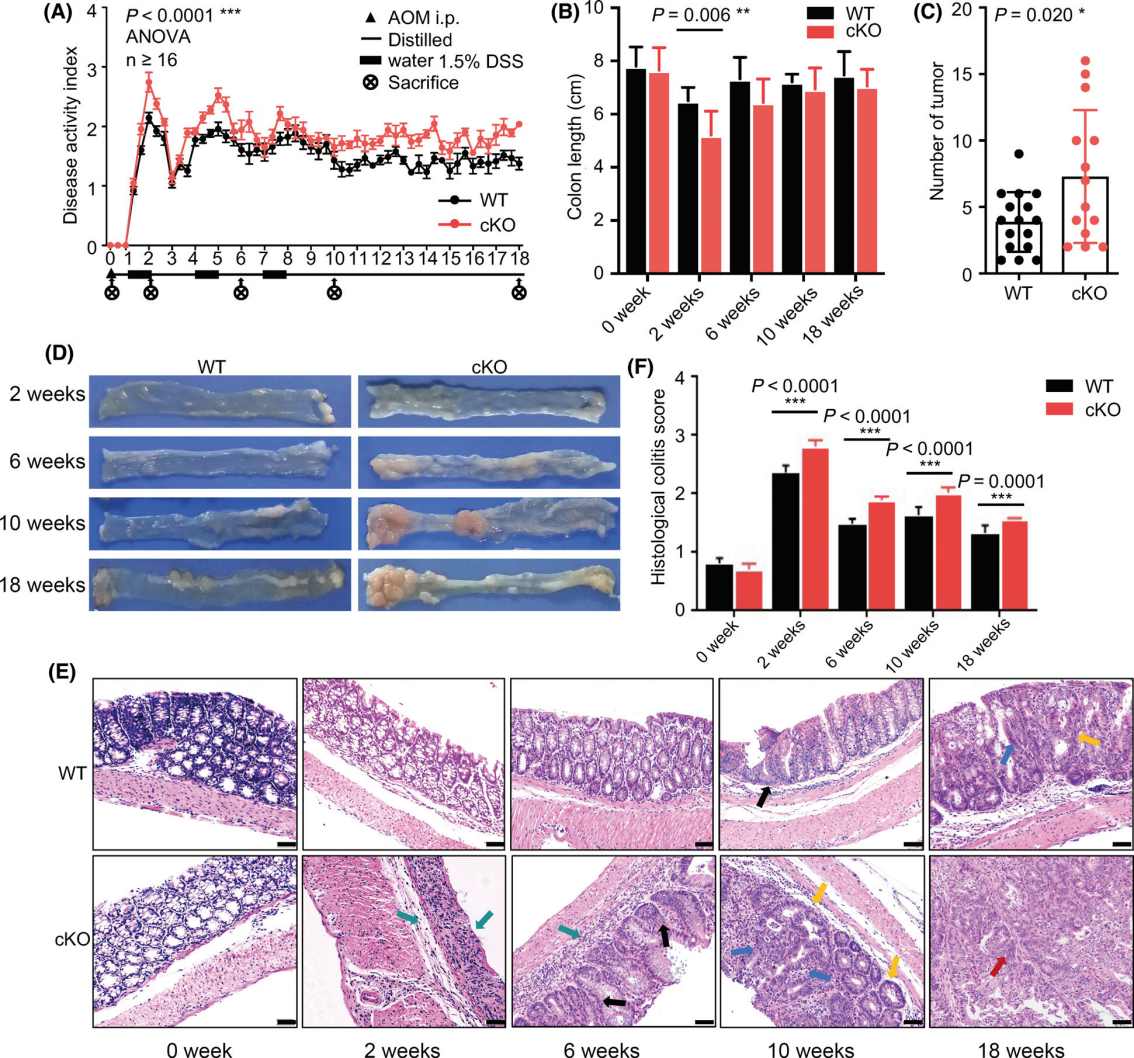
miR‐126 deficiency in IECs exacerbated CAC development. (A) Changes in the disease activity index (DAI) during AOM/DSS treatment in the wild‐type (WT) and miR‐126^ΔIEC^ (cKO) groups (WT *n* = 21, KO *n* = 16). DAI = (weight loss score + stool characteristic score + haematochezia score)/3. (B) Length of the colon from each group (WT *n* = 8, KO *n* = 6). (C) The numbers of tumours were measured at the 18th week of AOM/DSS administration (WT *n* = 16, KO *n* = 14). (D) Gross colon morphology at the 2nd, 6th, 10th and 18th weeks of treatment. Colons were opened longitudinally (*n* = 5). (E, F) Representative images of histological sections (magnification: 200×; scale bar = 50 μm) and histological colitis scores of mice in each group (WT *n* = 8, KO *n* = 7). Green arrows indicated inflammatory cells infiltrating in the mucosal and submucosa; black arrows indicated dysplasia crypt; yellow arrows indicated aberrant crypt foci; blue arrows indicated high‐grade intraepithelial neoplasia; red arrows indicated carcinoma polyp. Data are presented as the mean ± SEM (Panel A) or mean ± SD. Statistical analyses were performed using two‐way ANOVA with Geisser–Greenhouse correction for DAI comparison and multiple unpaired *t* tests for the remaining data (*P < 0.05; **P < 0.01; and ***P < 0.0001).

We established a CAC model in miR‐126^ΔIEC^ mice and measured histological changes at the end of the 0th, 2nd, 6th, 10th and 18th weeks after treatment to elucidate the function of miR‐126 during CAC development. During treatment, the mean DAI scores of miR‐126^ΔIEC^ mice were higher than those of their wild‐type (WT) littermates, which indicated several symptoms, such as higher weight loss, more severe diarrhoea and heavy bleeding in the intestine, in miR‐126^ΔIEC^ mice (Fig. 2A). Meanwhile, due to the repeated cycles of epithelial wounding and repair caused by DSS treatment, colitis causes a thickening of the muscularis mucosae with the accumulation of extracellular matrix that contributes to the shortening or stiffening of the colon [40]. Mice deficient in miR‐126 in IECs exhibited a decreased colon length at the 2nd week (Fig. 2B) and an increased number of macroscopically visible polyps and tumours in the colonic tissue at the 18th week compared with WT mice (Fig. 2C,D).

After 2 weeks of AOM/DSS treatment, miR‐126^ΔIEC^ mice displayed severe epithelial barrier damage. Goblet cells were lost and replaced by cryptitis, crypt abscesses, erosions and large numbers of inflammatory cells infiltrating the colonic mucosa and submucosa (Fig. 2E, indicated by green arrows). Six weeks after induction, besides infiltrated inflammatory cells (indicated by green arrows), we also observed inflammatory hyperplasia and dysplasia crypt (indicated by black arrows) in the colonic tissues of miR‐126^ΔIEC^ mice. In the 10th week, with the repeating DSS treatment and repair cycles, chronic relapsing‐remitting inflammation accelerated the development of dysplasia and aberrant crypt foci (indicated by yellow arrows). Eventually, it led to high‐grade intraepithelial neoplasia (indicated by blue arrows) and carcinoma polyp (indicated by red arrows) at the 18th week in miR‐126^ΔIEC^ mice (Fig. 2E). However, in the colonic tissues from WT mice, we observed milder epithelial barrier damage at the 2nd and 6th weeks, less dysplasia crypt (indicated by black arrows) at the 10th week and more diminutive carcinoma polyp at the 18th week compared to that in miR‐126^ΔIEC^ mice. Meanwhile, the mean histological colitis scores of miR‐126^ΔIEC^ mice were higher than those in WT mice (Fig. 2F). Thus, mice with a miR‐126 deficiency in IECs displayed severer colonic inflammation and exacerbated CAC development.

### 3.3 *CXCL12* is a direct target of miR‐126

**Fig. 3:**
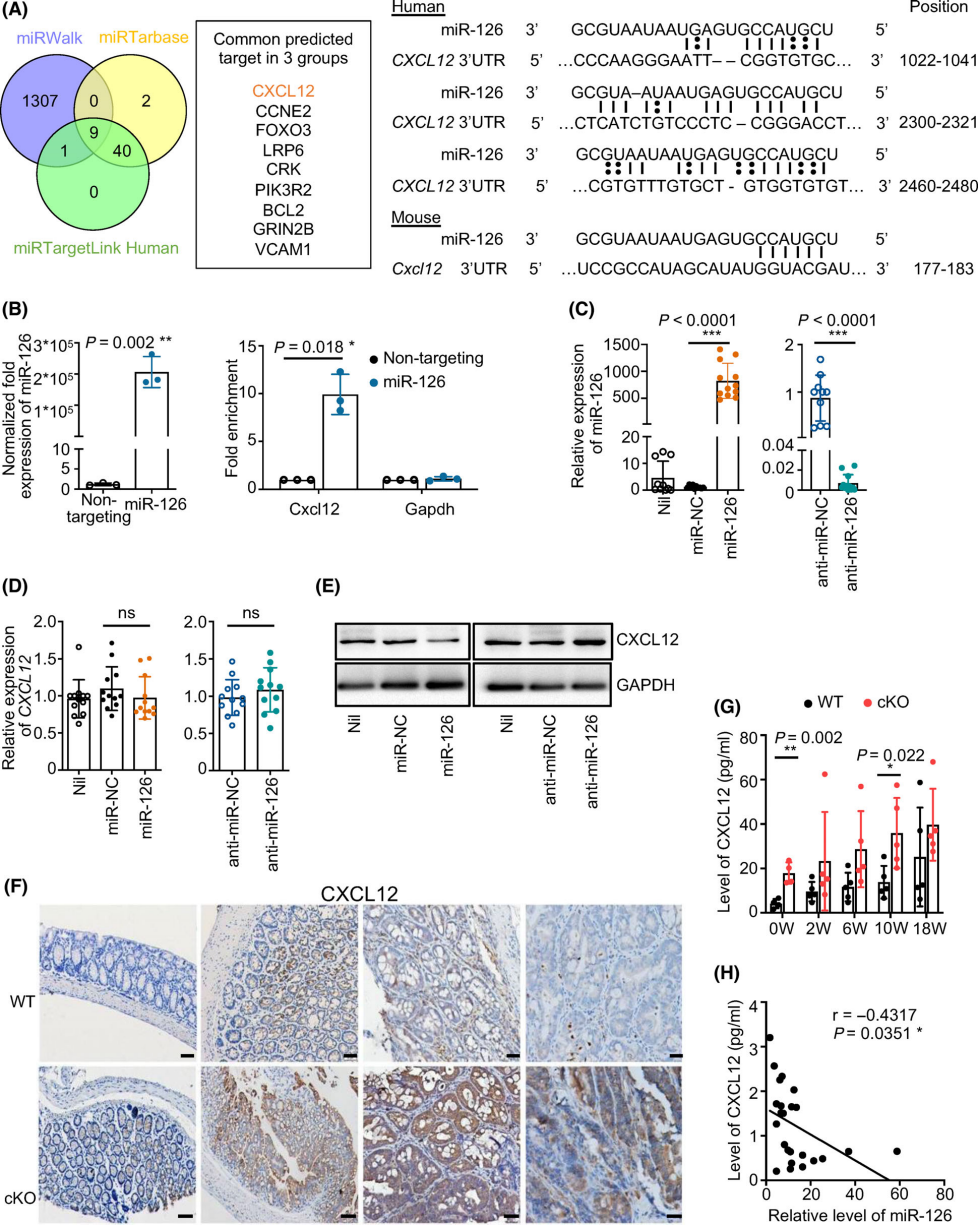
CXCL12 is a direct target of miR‐126. (A) Venn diagram of predicted targets showing that 1317, 51 and 41 targets were predicted by miRWalk, miRTarbase and miRTargetLink Human, respectively, and 9 candidate genes were predicted by all three algorithms (left panel). Binding sites between the CXCL12 3′UTR and miR‐126 were identified by miRanda and TargetScan (right panel). (B) The RNA pull‐down method was performed using miR‐126‐overexpressing HEK293 cells (left panel) and analysed the targeted regulation of CXCL12 by miR‐126 (right panel) (*n* = 3). (C) Establishment of miR‐126‐overexpressing and miR‐126‐silenced Caco2 cell lines. The relative expression of miR‐126 in these cell lines was analysed using qRT–PCR. U6 small nuclear RNA served as the internal qRT–PCR control (*n* = 10, 10, 12, 10 and 12, respectively). (D, E) Relative expression of CXCL12 in miR‐126‐overexpressing and miR‐126‐silenced Caco2 cell lines was analysed using qRT–PCR (*n* = 12) and western blotting (*n* = 3). (F, G) CXCL12 expression was assessed in colon tissues from the WT and cKO groups using IHC (magnification: 200×; scale bar = 50 μm) and ELISA (0 week, *n* = 4; 2, 6, 10 and 18 weeks, *n* = 5). (H) The correlation between the level of CXCL12 and the relative expression of miR‐126 in the colonic tissues from WT mice at 0, 2, 6, 10 and 18 weeks after induction was determined by the Pearson’s correlation test (*n* = 24). Individual data are presented as the mean ± SD. Statistical analyses were performed using unpaired *t* tests (*P < 0.05; **P < 0.01; and ***P < 0.0001).

A functional target is needed to further elucidate the mechanism by which miR‐126 protects against CAC tumorigenesis. We performed bioinformatics analyses to independently search for predicted targets of miR‐126 using the miRWalk, miRTarBase and miRTargetLinks databases. All candidate targets of miR‐126 are listed in Tables [Link], [Link], [Link]. Overlapping candidate targets were selected and listed (Fig. 3A, left panel). Among these targets, we focussed on CXCL12 as the only secreted protein that might recruit immune cells and interfere with the crosstalk between infiltrated immune cells and inflamed IECs (Table S4). We further predicted the miR‐126‐*CXCL12* interaction binding sites using miRanda and TargetScan. The algorithm predictions indicated that human miR‐126 has three potential binding sites (it targeted three evolutionarily conserved sequences) in *CXCL12*, whereas murine miR‐126 has one binding site (Fig. 3A, right panel). We further performed RNA immunoprecipitation to verify the binding between miR‐126 and *CXCL12*. As a first step in the binding analysis, lysates from HEK293 cells transfected with miR‐126 mimic were exposed to Protein G magnetic beads and pan‐Ago antibodies to capture RISC and RNA was further purified to detect downstream targets. As shown in the left panel of Fig. 3B, miR‐126 expression was significantly increased in the lysates after transfection compared with the nontargeting group, indicating the successful transfection of miR‐126 and the effective targeting of Protein G magnetic beads and pan‐Ago antibodies. In addition, the expression of the *CXCL12* mRNA in the pull‐down materials isolated from HEK293 cells transfected with miR‐126 was ten times greater than that in the control group (Fig. 3B, right panel), indicating that miR‐126 directly binds to the *CXCL12* mRNA while forming the RISC.

We further established miR‐126‐overexpressing and miR‐126‐silenced Caco2 colon cells (Fig. 3C) but did not observe a significant difference in *CXCL12* mRNA expression among the groups (Fig. 3D). When detecting the CXCL12 protein level, however, miR‐126 overexpression decreased CXCL12 production in colon cells, whereas miR‐126 silencing increased its level (Fig. 3E). In addition, we detected a higher level of CXCL12 in the colon tissues from miR‐126^ΔIEC^ mice than in those from WT mice and from patients with UC and CD, especially in CAC tissues (Fig. 3F,G, Fig. S2A,C). Furthermore, the expression of miR‐126 was significantly negatively related to the level of CXCL12 in the colonic tissues from both mouse and patients (Fig. 3H, Fig. S2D). Collectively, these results imply that miR‐126 regulated CXCL12 expression at the post‐transcriptional level.

### 3.4 AMD3100 ameliorates mucosal damage and delays the onset of dysplasia in miR‐126^ΔIEC^ mice

**Fig. 4:**
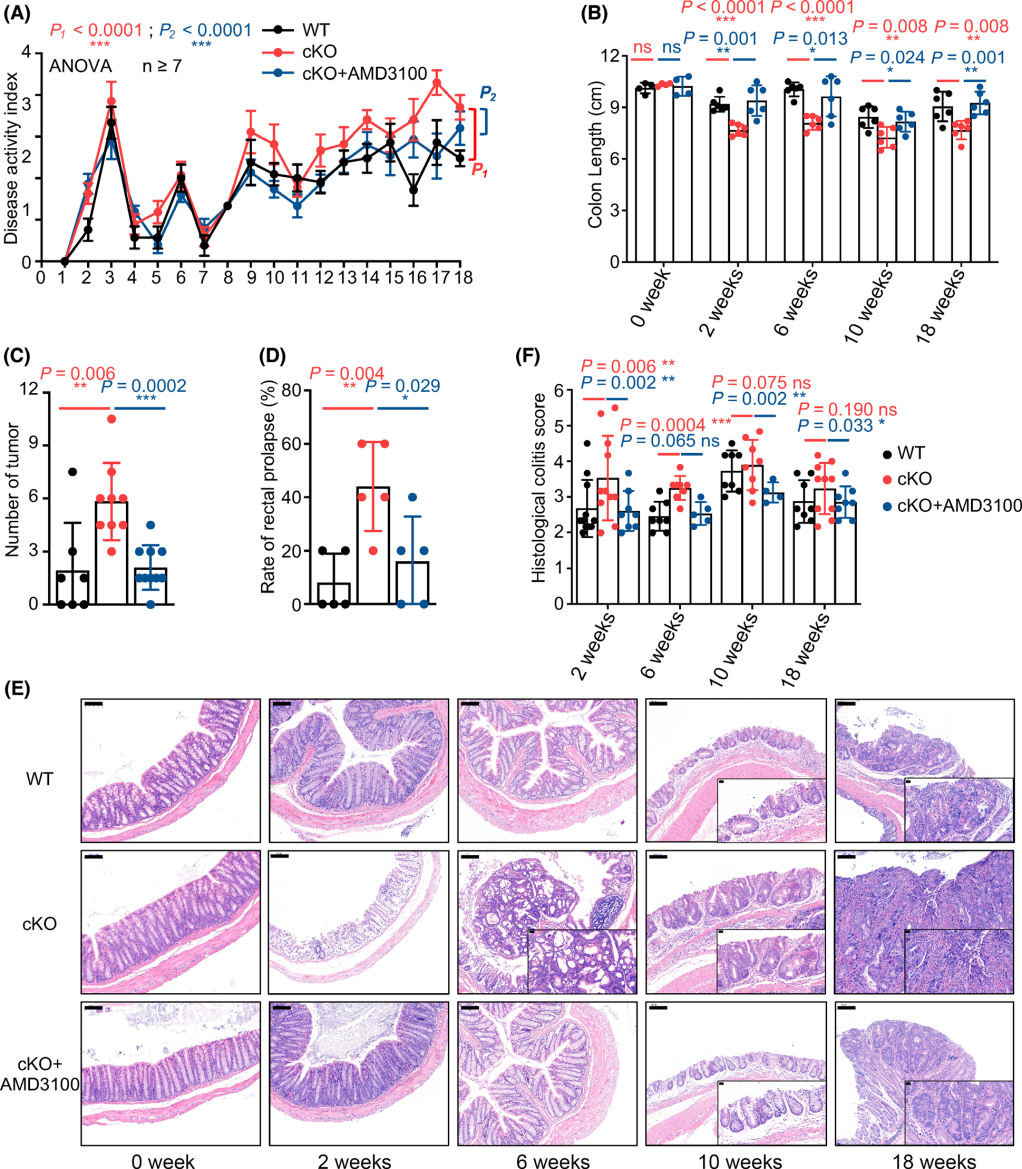
AMD3100 ameliorates mucosal damage and delays the onset of dysplasia in miR‐126^ΔIEC^ mice. (A) Changes in the disease activity index (DAI) during AOM/DSS treatment in the WT, miR‐126^ΔIEC^ and cKO+AMD3100 groups (WT, *n* = 7; cKO, *n* = 9; cKO+AMD3100, *n* = 10). (B) Length of the colon from each group (0 week, *n* = 4; 2, 6, 10 and 18 weeks, *n* = 6). (C) The numbers of tumours were measured at the 18th week of AOM/DSS administration (WT, *n* = 7; cKO, *n* = 9; cKO+AMD3100, *n* = 10). (D) Percentages of rectal prolapse in each group after the 10th week. (*n* = 5). (E, F) Representative images of histological sections (magnification: 100×; scale bar = 100 μm, inserts magnification: 400×; scale bar = 20 μm) and histological colitis scores of mice in each group (WT, *n* = 11, 8, 8 and 8, respectively; cKO, *n* = 11, 8, 8 and 10, respectively; cKO+AMD3100, *n* = 8, 5, 4 and 8, respectively). Data are presented as the mean ± SEM (Panel A) or mean ± SD. Statistical analyses were performed using two‐way ANOVA with Geisser–Greenhouse correction for DAI comparison and multiple unpaired *t* tests for the remaining data (*P < 0.05; **P < 0.01; and ***P < 0.0001).

According to previous studies, blockade of the CXCL12/CXCR4 axis by the specific antagonist AMD3100 attenuates experimental colitis in a murine model by inhibiting chronic inflammation and enhancing epithelial barrier integrity [41, 42]. We injected AMD3100 (5 mg·kg^−1^, i.p.) daily into miR‐126^ΔIEC^ mice following AOM/DSS treatment to determine the function of CXCL12 in miR‐126^ΔIEC^ mice during CAC development. We found that miR‐126^ΔIEC^ mice displayed an increased DAI score, decreased colon length and an increased number of tumour polyps compared with WT mice. AMD3100 treatment, however, alleviated colitis symptoms and reduced mean DAI scores in miR‐126^ΔIEC^ mice (cKO+AMD3100 group) compared with the miR‐126^ΔIEC^ mice that were not treated (cKO group) (Fig. 4A). Meanwhile, the colon length (Fig. 4B), number of tumour polyps (Fig. 4C) and rate of rectal prolapse (Fig. 4D) in the AMD3100‐treated mice also decreased compared to those in untreated mice.

The histological analysis revealed that AMD3100 attenuated severe inflammatory damage and early development of dysplasia and carcinomas in the colorectal tissues of miR‐126^ΔIEC^ mice (Fig. 4F). After one cycle of DSS treatment (at the 2nd week), miR‐126^ΔIEC^ mice displayed severe structural damage, including goblet cell loss, erosion and cryptitis, with substantial inflammatory cell infiltration in colonic tissues. However, AMD3100‐treated miR‐126‐deficient mice, which resembled WT mice, largely retained the integration of epithelial barriers. In addition, in the 6th week, we found a few dysplastic crypt loci in miR‐126^ΔIEC^ mice but rare in other groups. Hence, upon AOM/DSS treatment, AMD3100 attenuated the inflammatory damage in the mucosa and delayed the onset of dysplasia in miR‐126^ΔIEC^ mice.

### 3.5 miR‐126 inhibits macrophage recruitment via CXCL12

CXCL12 has been reported as a chemokine attracting macrophages, neutrophils and lymphocytes by binding to CXCR4. Using the TIMER database, we found that CXCL12 expression has significant positive correlations with infiltrating levels of macrophages, neutrophils, CD4+ T cells, CD8+ T cells and B cells (Fig. S3A). The correlations between the somatic copy number alteration of CXCL12 and the abundance of immune infiltrates determined that deep deletion of CXCL12 was significantly related to the decreased infiltration level of macrophages in COAD (Fig. S3B).

**Fig. 5:**
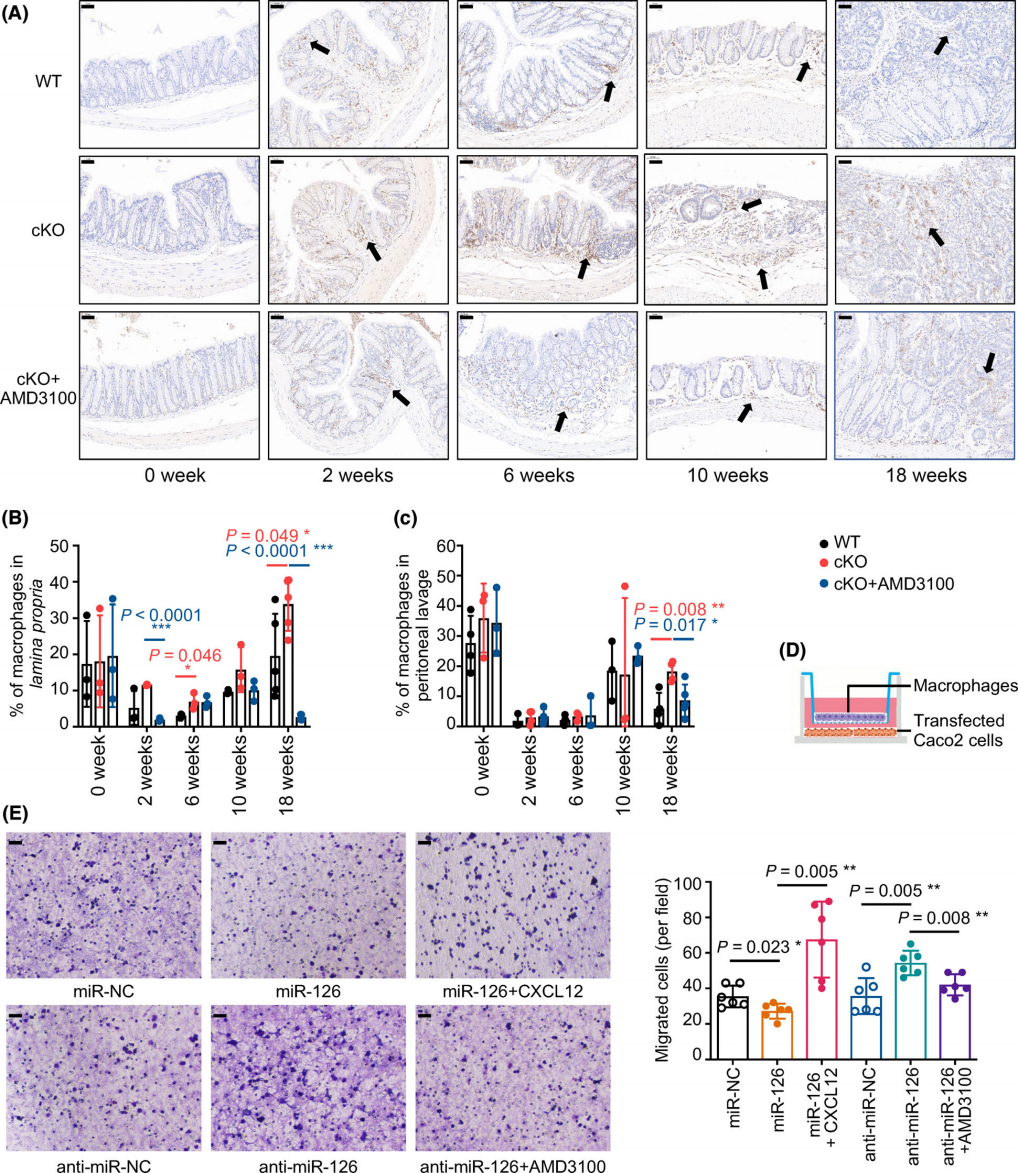
miR‐126 inhibits macrophage recruitment via CXCL12. (A) Representative images of immunohistochemical staining for CD68 in colonic mucosa in mice from each group at 0, 2, 6, 10 and 18 weeks after AOM/DSS treatment (magnification: 200×; scale bar = 50 μm, *n* = 4). Black arrows indicated positive cells. (B, C) Flow cytometry detection of colorectal macrophages in *lamina propria* or peritoneal lavage isolated from AOM/DSS mice at 0, 2, 6, 10 and 18 weeks after induction. Two‐parameter histograms of F4/80^+^CD11b^+^ were recognised as macrophages and quantified (*n* = 3, 3, 3, 3 and 4, respectively). (D) Model of the cell migration assay for macrophages. (E) Transwell assays detected the chemotaxis of macrophages by colon cancer cells overexpressing or lacking miR‐126 and the recruitment of macrophages by miR‐126 after CXCL12 or AMD3100 (100 ng·mL^−1^) stimulation (magnification: 200×; scale bar = 50 μm, *n* = 6). Individual data are presented as the mean ± SD. Statistical analyses were performed using unpaired *t* tests (*P < 0.05; **P < 0.01; and ***P < 0.0001).

In our study, after AOM/DSS induction, the amount of CD68+ macrophages significantly increased in the colonic lamina propria from mice of all groups at the 2nd, 6th, 10th and 18th weeks, especially in the miR‐126^ΔIEC^ mice (Fig. 5A, indicated by the black arrows). Moreover, the proportions of macrophages in the miR‐126^ΔIEC^ mice were increased compared with the WT mice but decreased in the AMD3100‐treated mice in either the peritoneal lavage or lamina propria, particular at the 18th week (Fig. 5B,C). Meanwhile, we found that the infiltrating level of Ly6G+ neutrophils significantly increased in the colonic tissues at the 2nd and 6th weeks, but there are no significant differences between WT, miR‐126^ΔIEC^ mice and AMD3100‐treated miR‐126^ΔIEC^ mice (Fig. S4A). In addition, the amount of CD4+ T cells, CD8+ T cells and PAX5+ B cells in the colonic tissues also slightly increased during the CAC process (Fig. S4B,C).

The inflammatory and tumour microenvironments play essential roles in regulating CAC progression, in which macrophages are significant components and play pivotal roles [43]. Therefore, we built a coculture system containing macrophages and miR‐126‐overexpressing or miR‐126‐silenced colon cells to detect its effect on macrophage recruitment and secretion *in vitro* (Fig. 5D). When cocultured with miR‐126‐overexpressing colon cells, the number of migrated TAMs was markedly decreased but increased when CXCL12 was added. Conversely, when cocultured with miR‐126‐deficient colon cells, the number of migrated macrophages was significantly increased, but the number decreased when the CXCL12/CXCR4 axis was blocked by AMD3100 (Fig. 5E). Thus, miR‐126‐overexpressing colon cells inhibited and miR‐126‐deficient colon cells promoted the recruitment of macrophages, and the CXCL12/CXCR4 axis mediated this regulatory effect. Taken together, miR‐126 regulates the recruitment of macrophages via CXCL12.

### 3.6 miR‐126 regulates cytokine levels via CXCL12

**Fig. 6:**
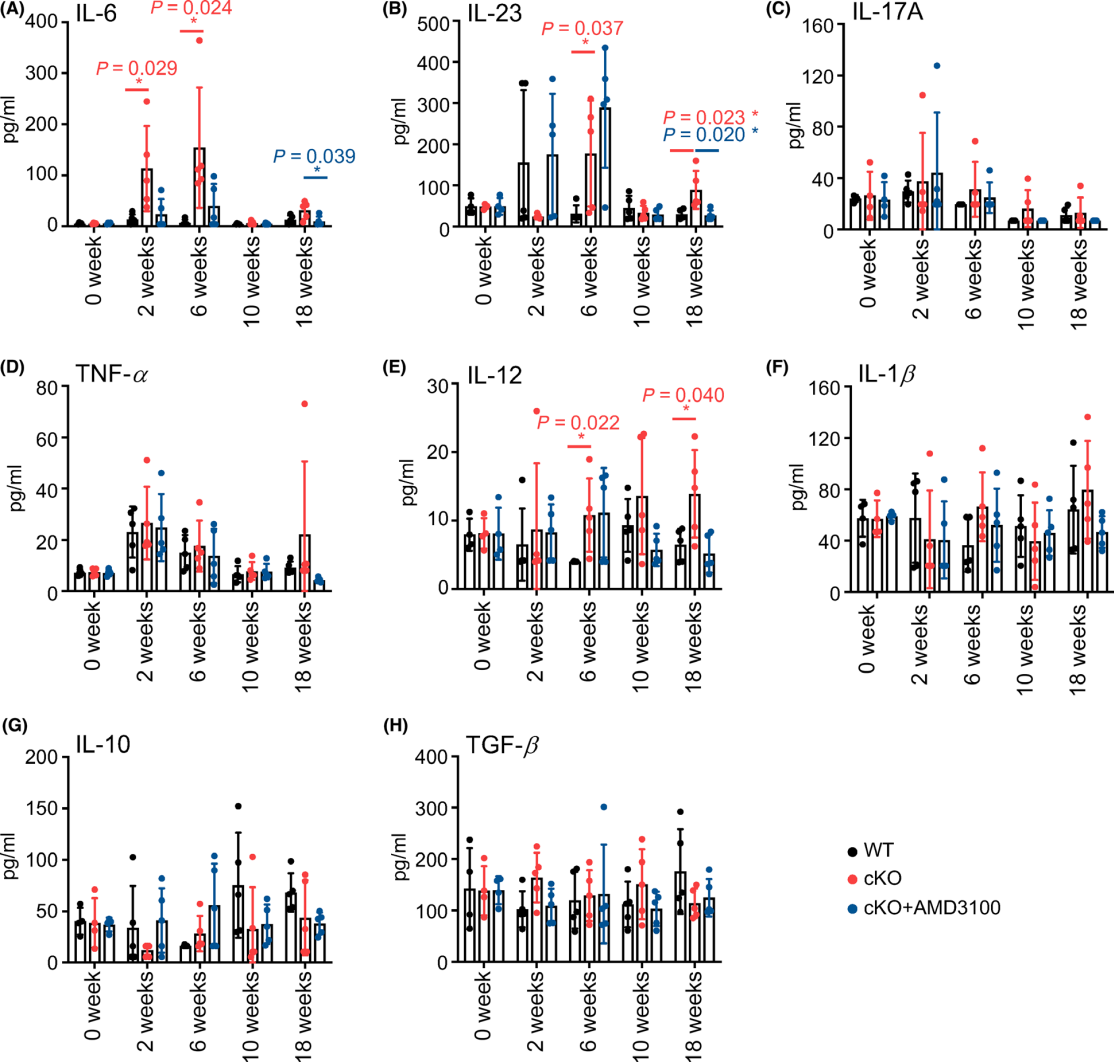
miR‐126 regulates cytokine levels via CXCL12. (A–H) Serum levels of IL‐6, IL‐23, IL‐17A, TNF‐α, IL‐12, IL‐1β, IL‐10 and TGF‐β were measured in mice from each group at 0, 2, 6, 10 and 18 weeks after AOM/DSS treatment (0 week, *n* = 4; 2, 6, 10 and 18 weeks, *n* = 5). Individual data are presented as the mean ± SD. Statistical analyses were performed using unpaired *t* tests (*P < 0.05).

The cytokine network is a key mediator of cellular interactions in the intestine under both physical and pathophysiological conditions. Impaired regulation of cytokine‐mediated interactions between epithelial cells and key orchestrators, such as recruited inflammatory macrophages, initiates and promotes chronic intestinal inflammation. By examining critical cytokines associated with the progression of IBD and CRC [19, 20], we detected increased serum levels of proinflammatory cytokines such as IL‐6, IL‐23, IL‐17A and TNF‐α in mice during the early stage of intestinal inflammation progression, namely, at the 2nd and 6th weeks (Fig. 6A–D). In contrast, IL‐12 and IL‐1β levels remained stable in the early stages but increased in the 10th and 18th weeks in miR‐126^ΔIEC^ mice (Fig. 6E,F). The level of the anti‐inflammatory cytokine IL‐10 increased at the 10th week, while TGF‐β levels remained stable during CAC progression (Fig. 6G,H). In addition, at different time points, the levels of the proinflammatory cytokines IL‐6 (at the 2nd and 6th weeks), IL‐12 (at the 6th and 18th weeks) and IL‐23 (at the 6th and 18th weeks) were markedly increased in miR‐126^ΔIEC^ mice compared to those in WT mice (Fig. 6A–C). AMD3100, however, decreased the elevated levels of IL‐6 and IL‐23 at the 18th week (Fig. 6A,C). In conclusion, miR‐126 deficiency in IECs resulted in increased serum levels of IL‐6, IL‐12 and IL‐23, whereas AMD3100 attenuated these changes during CAC development. These results imply that the regulation of cytokine production by miR‐126 partially depended on the CXCL12/CXCR4 axis.

### 3.7 Anti‐IL‐6 antibodies dampened the protumour activities of ‘educated’ macrophages towards colon cells

**Fig. 7:**
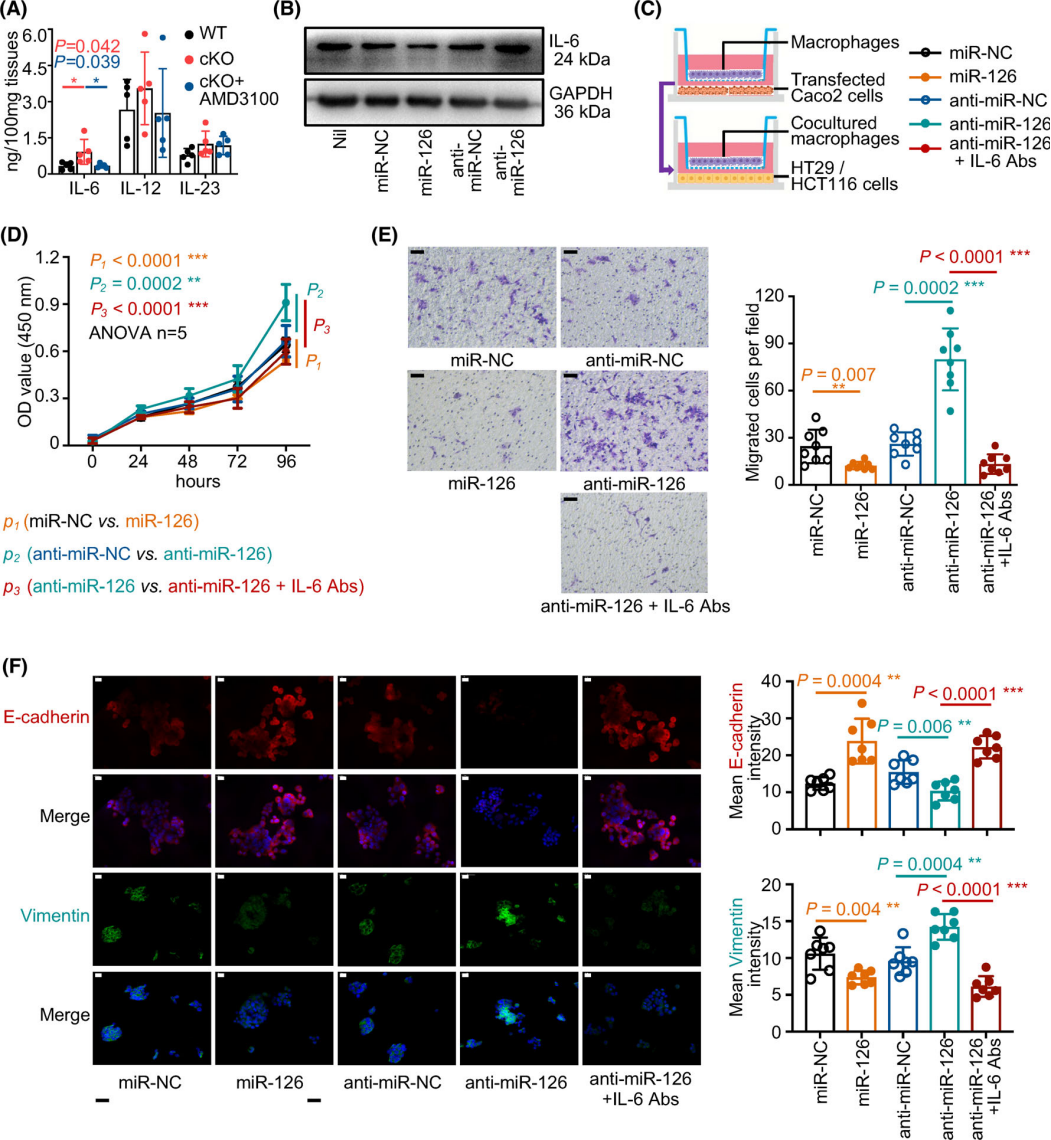
Anti‐IL‐6 antibodies dampened the protumour activities of ‘educated’ macrophages towards colon cells. (A)The level of IL‐6, IL‐12 and IL‐23 in the colonic tissues were measured in mice from each group at 18 weeks after AOM/DSS treatment using ELISA (*n* = 5). (B) Western blot analysis of IL‐6 levels in macrophages cocultured with miR‐126‐overexpressing or miR‐126‐silenced colon cells (*n* = 3). (C) Model of the coculture system of TAMs and colon cells. TAMs were first cocultured with miR‐126‐overexpressing or miR‐126‐silenced colon cells for 2 days. The cocultured TAMs were then removed from the previous system and placed in a new coculture system with untreated colon cells, and IL‐6 neutralising antibodies (Abs, 100 ng·mL^−1^) were subsequently added. (D) CCK‐8 assays were performed to determine cell growth (*n* = 5). (E) Transwell assays were performed to measure the cellular recruitment capacity, and the numbers of migrated cells were counted (right panel). Macrophages were cocultured with miR‐126‐overexpressing or miR‐126‐silenced colon cells, and IL‐6 neutralising antibodies were added (magnification: 200×; scale bar = 50 μm, *n* = 8). (F) Representative images of immunofluorescence staining for E‐cadherin (red), Vimentin (green) and DAPI (blue) (magnification: 400×; scale bar = 20 μm, *n* = 7, left panel). The mean immunofluorescence staining density of E‐cadherin (upper right panel) and Vimentin (lower right panel) was measured (magnification: 400×; scale bar = 20 μm, *n* = 7). Individual data are presented as the mean ± SD. Statistical analyses were performed using unpaired *t* tests (*P < 0.05; **P < 0.01; and ***P < 0.0001).

In the colonic tissues from mice of three groups at the 18th week after induction, we detected the level of IL‐6, IL‐12 and IL‐23 and found that the level of IL‐6 significantly increased in miR‐126^ΔIEC^ mice compared to that in WT mice, whereas AMD3100 treatment dampened the elevated levels of IL‐6 (Fig. 7A). Our previous study found that colorectal M1‐type macrophages express a high level of IL‐6 during CAC [44]. In the present study, we found that macrophages produced a low level of IL‐6 when cocultured with miR‐126‐overexpressing cells, but the expression increased when macrophages were cocultured with miR‐126‐silenced cells (Fig. 7B). These findings indicated that miR‐126‐overexpressing or‐silenced colon cells regulated the expression of IL‐6 in macrophages.

Colon cells stimulated with IL‐6 have displayed an increased epithelial‐mesenchymal transition (EMT) activity (the E‐cadherin level was reduced, and the Vimentin level was increased) (Fig. S5A) and an increased capacity for migration (Fig. S5B–E). In the current study, we established a two‐step coculture system of macrophages and colon cells (Fig. 7C) to investigate the effect of macrophage‐secreted IL‐6 on colon cells. In this system, we removed macrophages from the first coculture system, where macrophages were plated in the upper chamber and were cocultured with miR‐126‐overexpressing or miR‐126‐deficient colon cells in the lower chamber for 2 days and placed them in the second coculture system in which those cocultured ‘educated’ macrophages were cultured with untreated HT29 or HCT116 colon cells for another 2 days, and an IL‐6 neutralising antibody was added.

After we removed these ‘educated’ macrophages and cultured them with untreated colon cells, we found that macrophages ‘educated’ by miR‐126‐overexpressing cells inhibited and macrophages ‘educated’ by miR‐126‐silenced cells promoted the proliferation of colon cells. However, the IL‐6 neutralising antibody reduced the proliferation of these cells (Fig. 7D). Moreover, macrophages ‘educated’ by miR‐126‐overexpressing cells inhibited the migration of untreated colon cells and decreased their EMT activity by increasing E‐cadherin levels and decreasing Vimentin levels. Conversely, after coculture with miR‐126‐silenced cells, macrophages promoted colon cell migration and increased their EMT activity by reducing E‐cadherin levels and increasing Vimentin levels. Moreover, the IL‐6 neutralising antibody also attenuated these protumour activities (Fig. 7E,F), indicating that macrophages cocultured with miR‐126‐overexpressing or ‐silenced cells subsequently regulate colon cell proliferation and migration by secreting IL‐6.

## 4 Discussion

Despite the increasing attention paid to the crucial role of adaptive immunity in the development of IBD and CAC [45], macrophages are considered one of the essential components of innate immunity in the tumour microenvironment and play an essential role in maintaining intestinal homeostasis through the cell–cell interaction network [19]. Dysregulated interactions between IECs and macrophages and accompanying disequilibrium between proinflammatory and anti‐inflammatory cytokines have been implicated in the pathogenesis of CAC [24, 25]. As shown in the current study, miR‐126 expression decreased in patients with CRC and was significantly reduced during colorectal tumorigenesis in the experimental model. By generating miR‐126^ΔIEC^ mice to establish experimental models of CAC, we found that mice with a miR‐126 deficiency in IECs were more vulnerable to colitis‐associated tumorigenesis induced by AOM/DSS insults. We identified CXCL12 as a target gene of miR‐126 in inhibiting the development of colitis and CAC. Furthermore, miR‐126 regulated the recruitment of macrophages by targeting CXCL12 and inhibited the levels of proinflammatory cytokine IL‐6 in macrophages. Moreover, IL‐6 altered the proliferation and migration of IECs and might inhibit CAC tumorigenesis.

miRNAs play essential roles in maintaining gut homeostasis and regulating the development of inflammatory diseases and cancer [8, 9]. Impairment of global miRNA function enhances inflammation‐associated tumorigenesis in a mouse model [10]. When deregulated themselves, specific miRNAs regulate intestinal inflammation and CAC by modulating the expression of crucial molecular mediators linking inflammation and cancer [46]. For example, miR‐214 is overexpressed in the colonic tissues from patients with long‐lasting colitis and CAC, and it reduces PDLIM2 and PTEN expression in human colonocytes, increases the phosphorylation of AKT and activates nuclear factor‐kB, which induces IL‐6 expression and forms a feedback loop that subsequently amplifies the inflammatory response and promotes CRC development [47]. In contrast, miR‐26a targets and suppresses IL‐6 expression in macrophages, further inhibiting oncogene activation in IECs and reducing their proliferation, therefore suppressing tumour formation in a mouse model [48]. In addition, other miRNAs, such as miR‐301A, miR‐146a and miR‐21, have all been reported to regulate colitis‐associated tumorigenesis [49, 50, 51].

The current study demonstrated a significantly decreased miR‐126 expression in the colonic tissues in the CAC mouse model and in carcinoma tissues from CRC patients. In the colonic tissues from UC and CD patients, the expression range of miR‐126 is broad, and no significant difference was observed between colonic tissues from IBD patients and control or CRC patients. Some research reported an increased miR‐126 expression in colon tissues from patients with IBD [52, 53]. This inconsistency indicates a difference between research that might be interfered with sample size, sample types (endoscopic biopsies versus surgical specimens) or detecting methods (ISH versus PCR). Our previous research suggested that miR‐126 inhibits the proliferation of colon cancer cells, migration, invasion and metastasis [14, 15, 17]. In *in vivo* experiment, miR‐126 inhibits the tumorigenicity and metastasis of colon cancer cells in nude mice [17]. Consistent with previous studies [54, 55], we found that mice lacking miR‐126 in IECs exhibited enhanced colitis and dysplasia induced by AOM/DSS insults, which indicated a protective role for miR‐126 expressed in IECs in the development of CAC.

Our study identified *CXCL12* as a target gene of miR‐126 that regulates macrophage functions. CXCL12, binding to its receptor CXCR4, has been reported to be related to the prognosis of several types of cancer and promote cancer progression in preclinical models, including IBD and CRC [56, 57, 58]. The expression of CXCL12 and its ligand CXCR4 are independent prognostic factors for the 5‐year disease‐free survival rate of patients with colon cancer [59]. Blocking the CXCL12/CXCR4 axis ameliorates experimental colitis in a murine model, indicating a crucial role in the intestinal inflammatory response [41, 42]. Recent studies have documented constitutive CXCR4 expression in IECs and LPLs, and its expression is increased in IECs from patients with IBD [60]. Consistent with the finding of increased serum CXCL12 levels [26], we also observed increased CXCL12 levels in the colonic tissues during the ‘inflammation‐dysplasia‐cancer’ process, especially in miR‐126^ΔIEC^ mice.

Macrophages, commonly called tumour‐associated macrophages (TAMs) within the tumour, have been reported participating in crosstalk with cancer cells, facilitating CRC growth, progression and aggressiveness and correlating with the poor prognosis of patients with CRC [61, 62, 63]. CXCL12 is a potent chemotactic factor that recruits macrophages [64, 65], induces macrophages to release HE‐EGF and triggers anti‐apoptotic and proliferative signalling to promote cancer cell survival and expansion [66]. Consistent with previous findings [44, 67], our current study observed a significantly increased amount of CD68+ macrophages in the colonic lamina propria after AOM/DSS treatment. Meanwhile, the infiltrating level of Ly6G+ neutrophils significantly increased at the 2nd and 6th weeks, whereas the amount of infiltrated CD4+ T cells and CD8+ T cells increased slightly at a later stage of CAC (after 6 weeks upon induction). Thus, even though the total number of macrophages increased upon induction, its proportion decreased at the 2nd and 6th weeks.

Mice deficient in miR‐126 in IECs exhibited higher proportions of macrophages, but AMD3100 treatment damped the increase. In addition, we found that miR‐126‐overexpressing colon cancer cells inhibited macrophage recruitment, whereas miR‐126‐silenced cells promoted macrophage recruitment, and the CXCL12/CXCR4 axis mediated the regulatory effects. These findings implied CXCL12 as an essential mediator of the communication between colon cancer cells and macrophages during CAC.

Macrophages play a pivotal role in the host innate immune response to pathogen infections and are involved in tissue homeostasis, inflammation and cancer [23]. As one of the dominant cell types in the inflamed intestine, macrophages produce various cytokines, including IL‐6, IL‐12, IL‐23, IL‐1β and TNF, which modulate IEC activity and immune responses and evolve with and provide support to tumour cells during the transition to malignancy [24, 25]. In the present study, we found that the levels of proinflammatory cytokines such as IL‐6, IL‐23, IL‐17A and TNF‐α were increased during the progression of intestinal inflammation. Moreover, the levels of the proinflammatory cytokines IL‐6, IL‐12 and IL‐23 were markedly increased in miR‐126^ΔIEC^ mice compared to those in WT mice but reduced by AMD3100 treatment.

IL‐6 and the heterodimeric cytokines IL‐12 and IL‐23 are members of the class I haematopoietic family of cytokines based on their shared four‐a‐helix‐bundle motif and the homology within the structural domains of their receptors. The binding of these cytokines to their receptors activates the JAK‐STAT signalling pathway [68]. Multiple cell types in the tumour microenvironment, such as TAMs, granulocytes and fibroblasts, produce IL‐6, leading to JAK/STAT3 signalling activation in tumour cells that promotes cell proliferation, survival, invasiveness and metastasis [69].

Previous studies detected high IL‐6 expression in colorectal M1‐type macrophages during the ‘developing carcinoma’ and ‘metastasis’ processes in the CAC mouse model [44]. In addition, the IL‐6 downstream STAT3 signalling pathways are persistently activated during colitis‐associated carcinogenesis [26]. In the current study, we detected an increased level of IL‐6 in miR‐126^ΔIEC^ mice in the colonic tissues, whereas AMD3100 treatment dampened the elevation. In addition, miR‐126‐overexpressing or ‐silenced colon cells regulated the expression of IL‐6 in macrophages. CXCL12 has been reported to increase IL‐6 expression in fibroblasts and human basal cell carcinoma, while AMD3100 inhibits the CXCL12‐induced IL‐6 expression [70, 71]. In addition, serum IL‐6 levels are decreased in AMD3100‐treated mice [72]. Here, our study suggests that miR‐126 in CRC cells regulated the expression of IL‐6 in macrophages via the CXCL12/CXCR4 axis.

IL‐6 is a prototypical protumorigenic cytokine that contributes to the initiation and progression of colitis‐associated tumorigenesis by promoting cell progression, survival, tumour invasion and metastasis [69, 73]. Increased IL‐6 expression is related to an advanced stage of CAC and decreased survival of patients with CRC [74]. By establishing a two‐step coculture system of macrophages and colon cells, we demonstrated that macrophages, which were regulated by cocultured miR‐126‐transfected CRC cells, altered the proliferation and migration of colon cells via secreting IL‐6. These data thereby indicate that miR‐126 affects the function of macrophages and subsequently inhibits colon cell proliferation and migration via CXCL12/IL‐6 signalling.

The inflammation‐to‐carcinoma transition is an interesting and complex topic. The exact function of immune cells or specific treatment is dependent on the CAC stage and its microenvironmental context. One research demonstrated that the treatment with the intestinal helminth *Heligmosomoides polygyrus* at the onset of tumour progression in a mouse model of CAC does not alter tumour growth and distribution, but infection in the early inflammatory phase of CAC strengthens the inflammatory response and significantly boosts tumour development [75]. Our study has observed a transition from chronic inflammation to dysplasia and high‐grade intraepithelial neoplasia in the colonic tissues of mice around 6–10 weeks after AOM/DSS induction for miR‐126^ΔIEC^ mice. AMD3100, blocking CXCL12/CXCR4 axis, ameliorates mucosal damage and delays the onset of dysplasia in miR‐126^ΔIEC^ mice.

Our study suggests that miR‐126 affects the function of macrophages, subsequently suppresses epithelial transformation and ultimately inhibits tumour development via CXCL12/IL‐6 signalling. Nevertheless, considering the complex cell–cell interaction network in the mucosal milieu at different CAC stages, it is not sufficient to only target miR‐126 in the entire network system. It is necessary to establish a precise research model and explore the explicit role of miR‐126 in this network at a certain stage. For example, knockout miR‐126 or IEC‐specific CXCL12 or macrophages‐specific IL‐6 in a specific stage between inflammation and dysplasia. Thus, explicit study on miRNAs, targets, cell–cell interaction networks in multiple time points will provide us with the opportunity to intervene in early stage and delay tumour progression.

## 5 Conclusions

In summary, miR‐126 exerts an antitumour effect on CAC by regulating the crosstalk between IECs and macrophages via the CXCL12/IL‐6 axis. To our knowledge, this study is the first to focus on the regulation of CXL12 and IL‐6 expression on macrophages and its effect on IECs in CAC, raising the possibility that a range of complicated crosstalk events between IECs and immune cells may exist that are regulated by miRNAs and function through chemokines and cytokines, in addition to direct cell‐cell interactions. Our findings provide a potential practical method to inhibit macrophage‐mediated proinflammatory pathways, considering the extraordinary efforts made to develop microRNA therapeutics [76], which may have significant therapeutic potential for preventing/treating chronic colitis and CRC. In addition, more colonic tissues from patients with IBD‐associated CAC are needed to confirm the regulatory network between IECs and immune cells in inflamed and dysplastic intestines. Furthermore, future studies determining the efficacy of delivering miR‐126 to attenuate CAC development will be required to determine the therapeutic potential.

## Conflict of interest

The authors declare no conflict of interest.

## Author contributions

XYW and SW contributed to the conception and design of the study. SW performed most of the experiments and analysis and drafting of the manuscript. WY contributed to the design and performed part of the work on inducting CAC in miR‐126^ΔIEC^ mice. WWL performed IHC detection in colon tissues from mice. KN contributed to sample acquisition. XW, XRM and ZHS participated in the analysis and discussion of the results. XYW supervised the project and revised the manuscript. All authors contributed to manuscript revision, read and approved the submitted version.

## Supporting information


**Fig. S1.** Establishment of miR‐126^ΔIEC^ mice.

**Fig. S2.** Expression of miR‐126 and CXCL12 in the colonic mucosa from patients.

**Fig. S3.** Analysis of infiltrating immune cells.

**Fig. S4.** The infiltrating immune cells in the colonic tissues during CAC process.

**Fig. S5.** IL‐6 increases colon cells’ EMT activity and promotes its metastasis.

**Table S1.** The miRNA‐mRNA interaction pairs predicted by the miRWalk2.0 database.

**Table S2.** The miRNA‐mRNA interaction pairs predicted by the miRTarBase database.

**Table S3.** The miRNA‐mRNA interaction pairs predicted by the miRTargetLink Human database.

**Table S4.** Details of overlapping miR‐126 candidate targets provided by The Human Protein Atlas.

**Table S5.** The detailed profile of cells used in this study.

## Data Availability

The data that support the findings of this study are openly available in the GSE115513 at https://www.ncbi.nlm.nih.gov/geo/query/acc.cgi?acc=GSE115513 [31].
