# Supplementary figures and images for: miR‐126 downregulates CXCL12 expression in intestinal epithelial cells to suppress the recruitment and function of macrophages and tumorigenesis in a murine model of colitis‐associated colorectal cancer
Source: Mol Oncol. 2022 Apr 11;16(19):3465–89. doi: 10.1002/1878-0261.13218 (PMC9533691; doi:10.1002/1878-0261.13218)

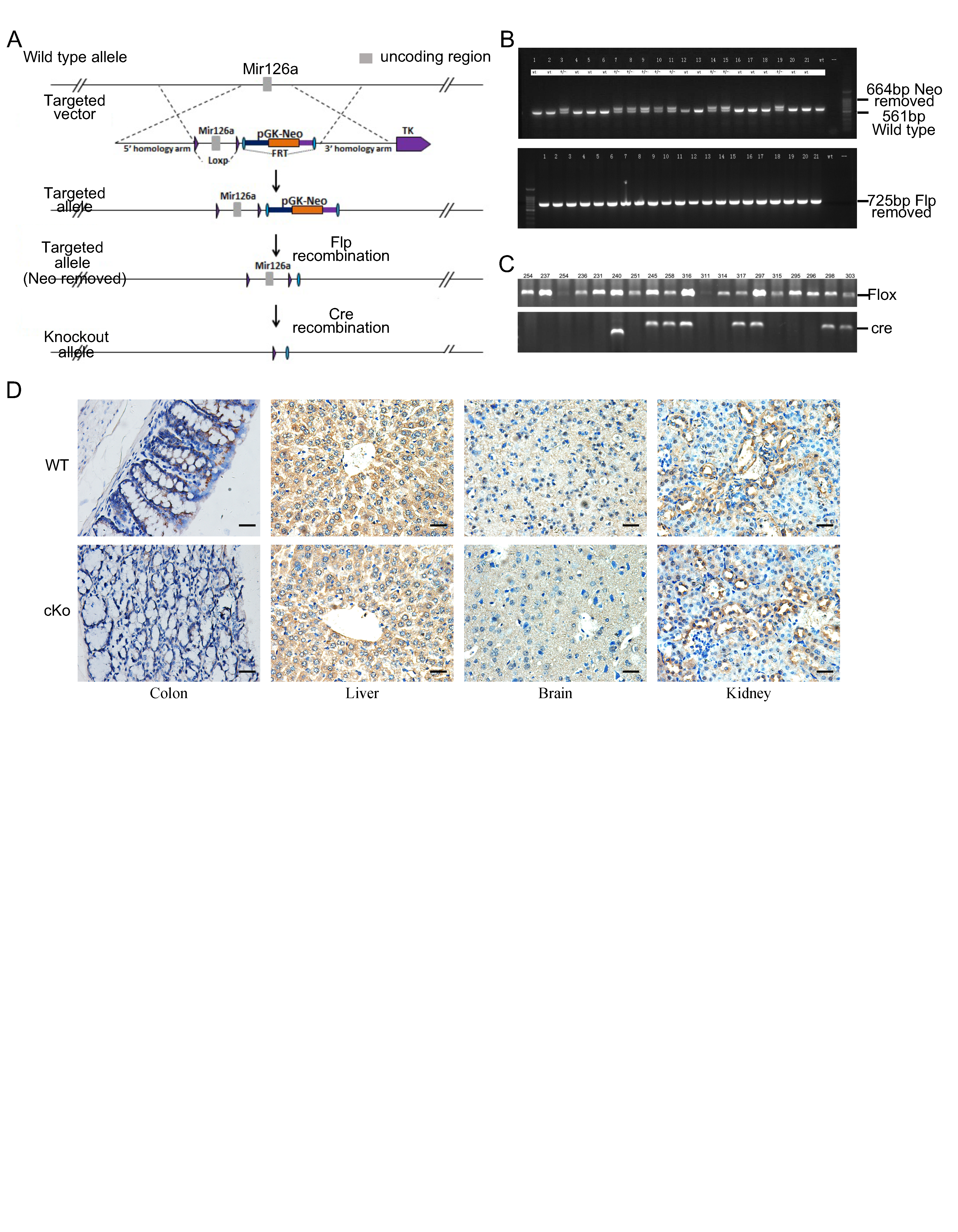

Supplement: Supplementary file 1 — Fig. S1. Establishment of miR‐126ΔIEC mice. [file MOL2-16-3465-s006.tiff]

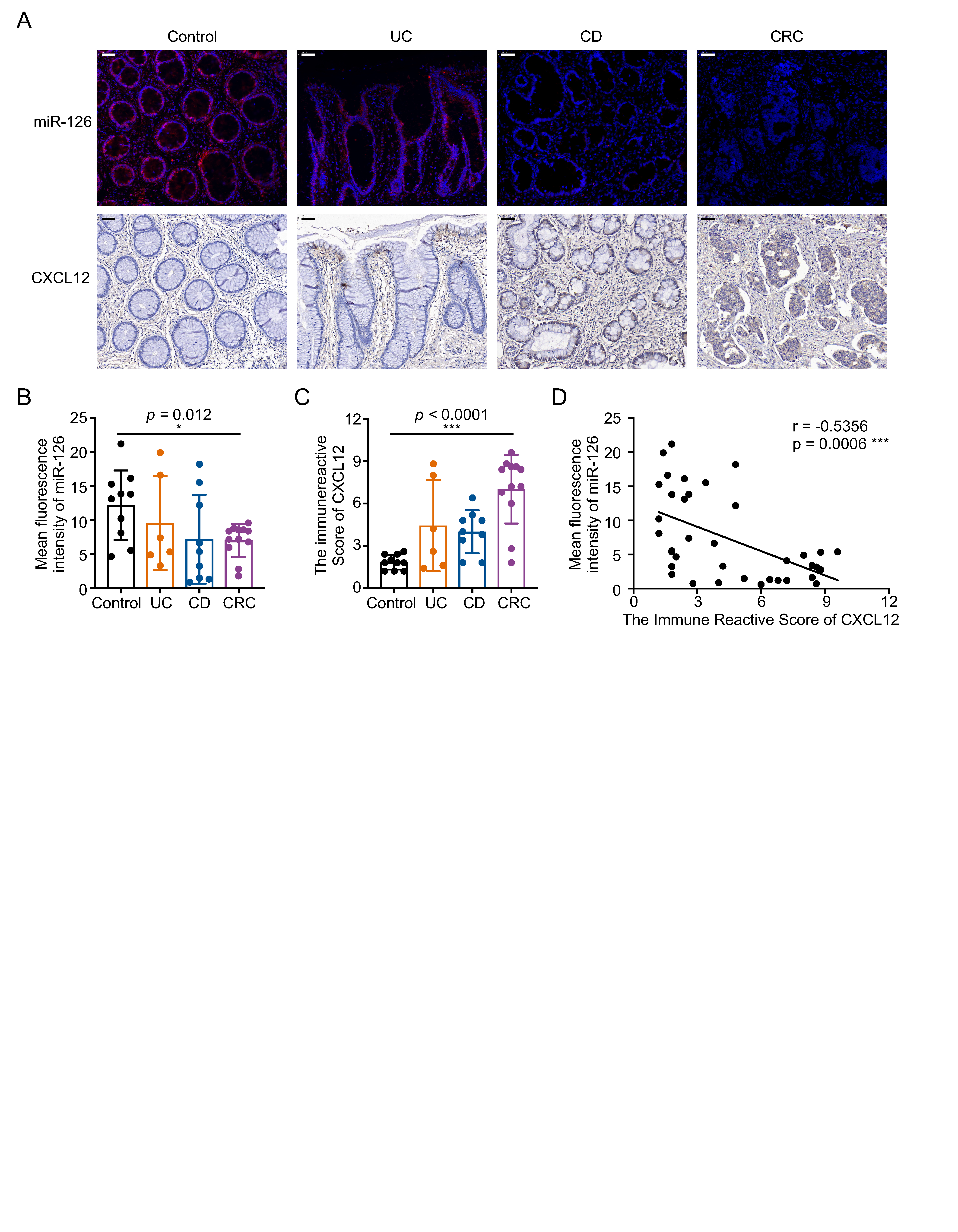

Supplement: Supplementary file 2 — Fig. S2. Expression of miR‐126 and CXCL12 in the colonic mucosa from patients. [file MOL2-16-3465-s007.tiff]

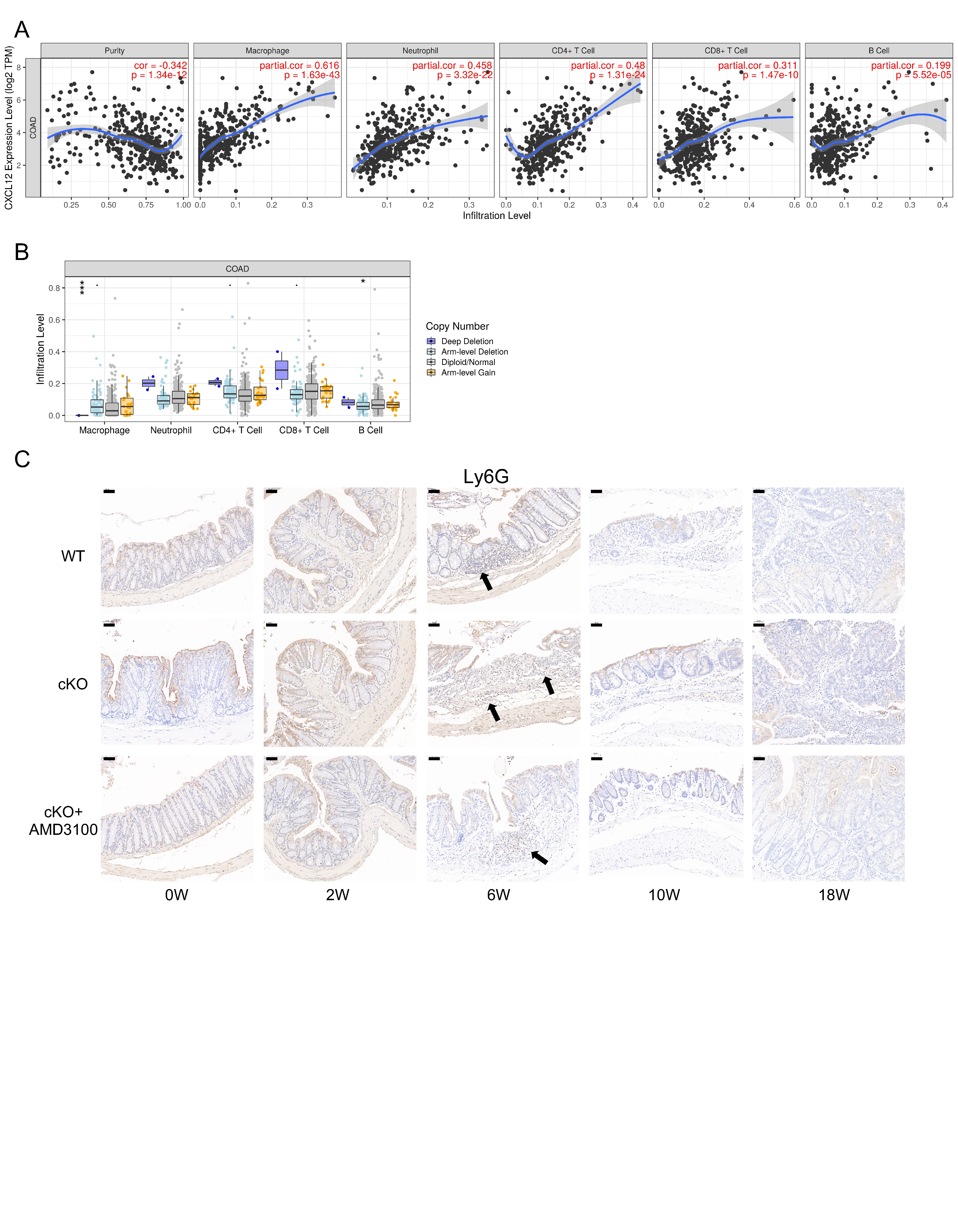

Supplement: Supplementary file 3 — Fig. S3. Analysis of infiltrating immune cells. [file MOL2-16-3465-s010.tiff]

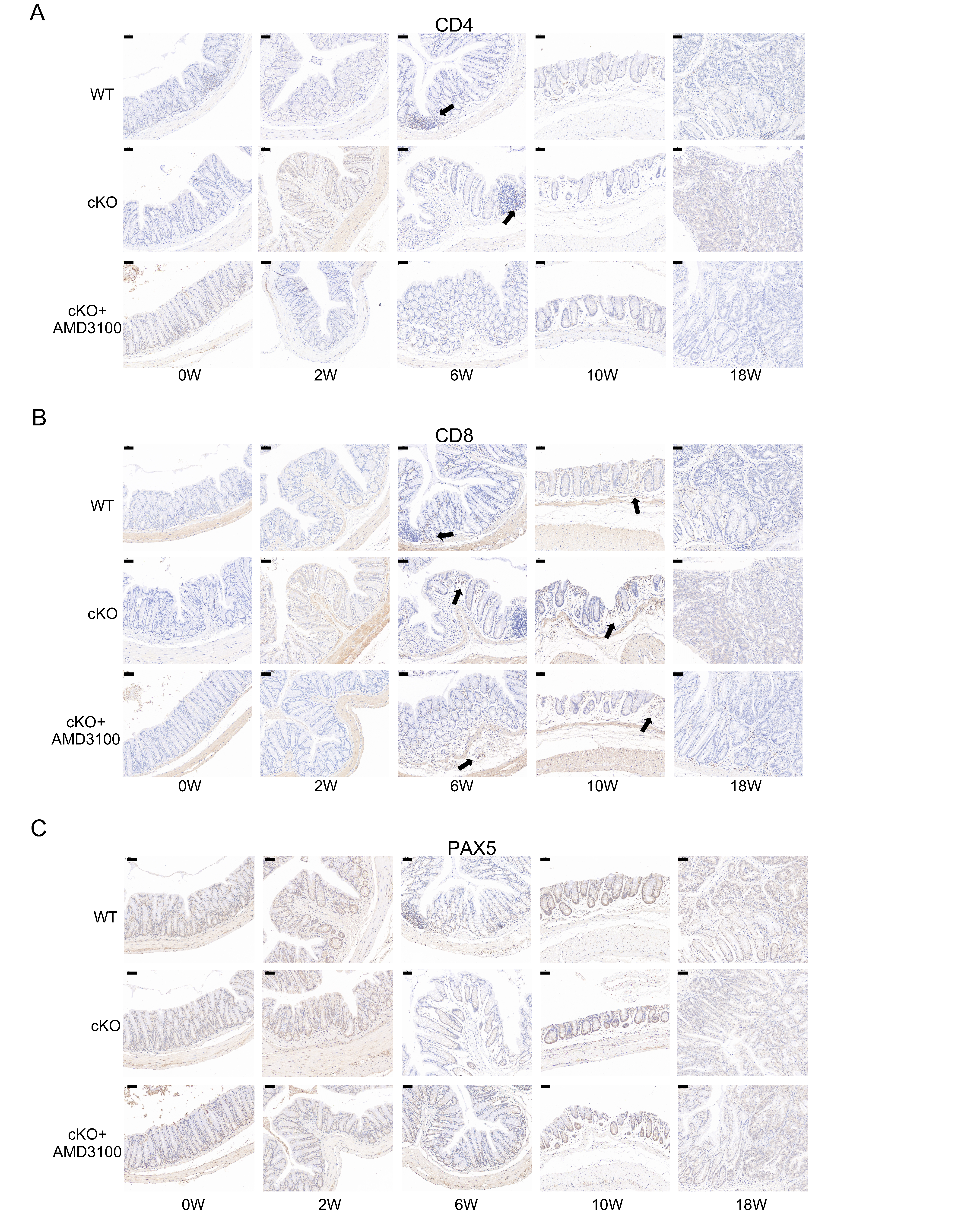

Supplement: Supplementary file 4 — Fig. S4. The infiltrating immune cells in the colonic tissues during CAC process. [file MOL2-16-3465-s004.tiff]

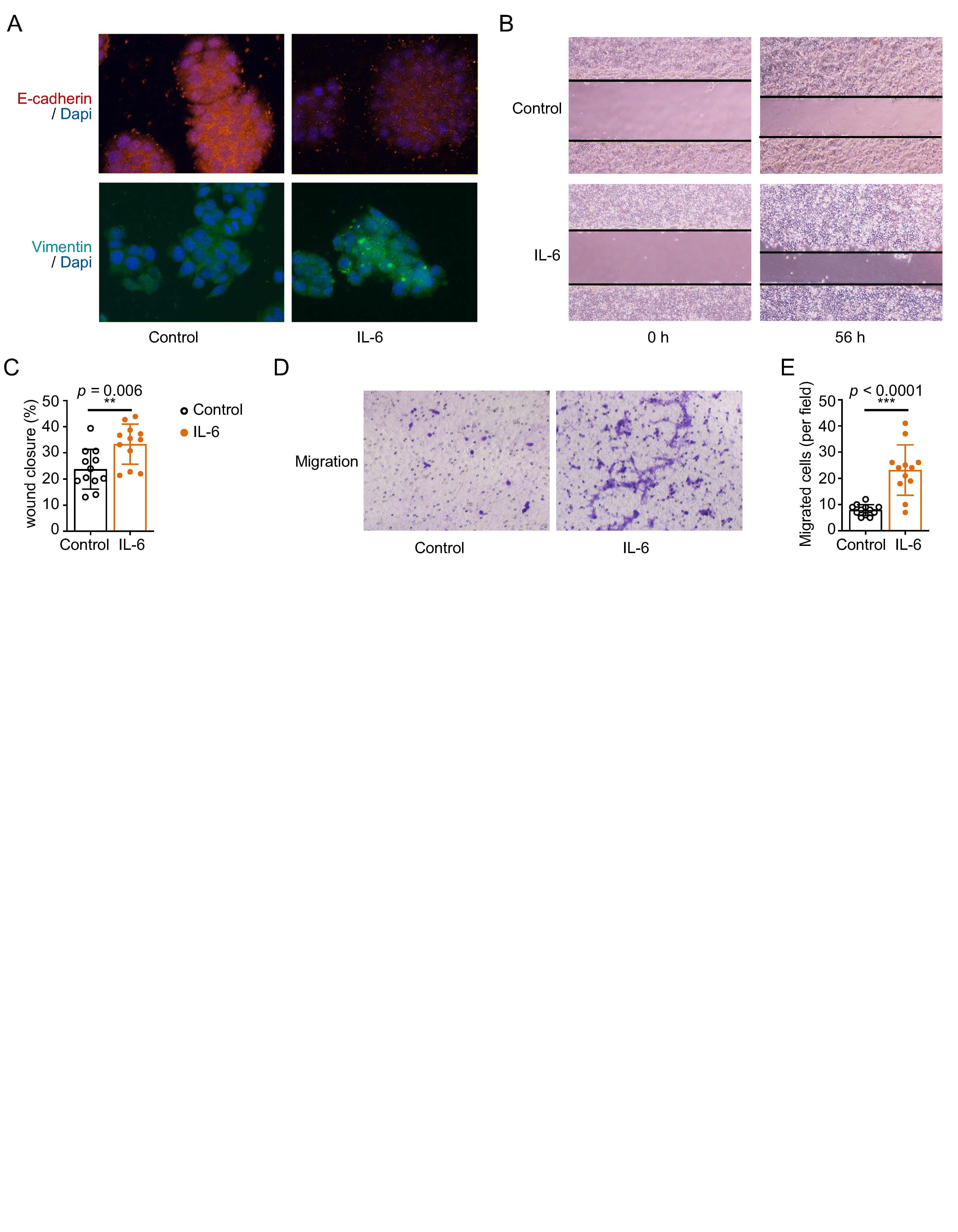

Supplement: Supplementary file 5 — Fig. S5. IL‐6 increases colon cells’ EMT activity and promotes its metastasis. [file MOL2-16-3465-s008.tiff]
